# Environment-independent distribution of mutational effects emerges from microscopic epistasis

**DOI:** 10.1101/2023.11.18.567655

**Published:** 2023-11-18

**Authors:** Sarah Ardell, Alena Martsul, Milo S. Johnson, Sergey Kryazhimskiy

**Affiliations:** 1 Department of Ecology, Behavior and Evolution, University of California San Diego, La Jolla, CA 92093; 2 Department of Integrative Biology, University of California Berkeley, Berkeley, CA 94720; 3 Biological Systems and Engineering Division, Lawrence Berkeley National Laboratory, Berkeley, CA, USA.

## Abstract

Predicting how new mutations alter phenotypes is difficult because mutational effects vary across genotypes and environments. Recently discovered global epistasis, where the fitness effects of mutations scale with the fitness of the background genotype, can improve predictions, but how the environment modulates this scaling is unknown. We measured the fitness effects of ~100 insertion mutations in 42 strains of *Saccharomyces cerevisiae* in six laboratory environments and found that the global-epistasis scaling is nearly invariant across environments. Instead, the environment tunes one global parameter, the background fitness at which most mutations switch sign. As a consequence, the distribution of mutational effects is remarkably predictable across genotypes and environments. Our results suggest that the effective dimensionality of genotype-to-phenotype maps across environments is surprisingly low.

## One Sentence Summary

The effects of mutations on microbial growth rate follow a pattern of global epistasis that is invariant across environments.

Adaptive evolution can lead to profound changes in the phenotypes and behaviors of biological systems, sometimes with adverse and sometimes with beneficial consequences for human health, agriculture and industry (1–5). However, predicting these changes remains difficult (6, 7). One major challenge is that how new mutations alter phenotypes and fitness of organisms often depends on the genetic background in which they arise (G×G interactions or “epistasis”), the environment (G×E interactions), or both (G×G×E interactions) (8). These interactions can alter not only the magnitude but also the sign of mutational effects, causing evolutionary trajectories to become contingent on the initial genotype, environment and random events (9–11).

Much of prior empirical work measured the effects of individual mutations on fitness-related phenotypes and characterized how these effects vary across genetic backgrounds and environments (9, 12–21). These studies of “microscopic” G×G, G×E and G×G×E interactions provide important insights into the structure of empirical fitness landscapes and their evolutionary navigability (22–24). However, predicting evolution at the genetic level using this approach is difficult because the number of potential interactions grows super-exponentially with the number of variable loci (25–27). Predicting evolution at the phenotypic level may be more feasible and in many cases more useful (27, 28). Such predictions rely on coarse-grained, or “macroscopic” descriptions of G×G, G×E and G×G×E interactions that inform us about how the distributions of mutational effects change across genotypes and environments (22, 29–31). While the distributions of effects of mutations on fitness, or “DFEs”, have been measured in many systems (32–35), we lack a systematic understanding of how DFEs vary across genetic backgrounds and environments (31, 36).

Several recent studies have shown that many mutations tend to make the phenotype of an organism in which they occur less extreme (13, 37–46), an instance of a more general phenomenon of “global epistasis” (8, 47). Global epistasis is expected to arise for complex traits, including fitness (48), and it can be used to quantitatively predict the effects of individual mutations in new genetic backgrounds without the full knowledge of microscopic G×G interactions, thereby alleviating the combinatorial problem mentioned above (47, 48). More importantly, if most mutations exhibit global epistasis, the distributions of their phenotypic effects should also have a quantitatively predictable shape (48), which could facilitate evolutionary predictions at the phenotypic level. However, as the variation in these distributions remains poorly characterized, no attempt has been made so far to link macroscopic G×G, G×E and G×G×E interactions to the underlying models of microscopic global epistasis.

Probing whether global epistasis models can capture G×G, G×E and G×G×E interactions at both microscopic and macroscopic levels hinges on measuring the effects of many mutations across multiple genetic backgrounds and environments. To this end, we measured how ~100 quasi-random barcoded insertion mutations constructed in our previous study (42) affect growth rate in 42 “background” strains of yeast *Saccharomyces cerevisiae* in six conditions (see Methods). All background genotypes are segregants from a cross between two strains of yeast (RM, a vineyard strain, and BY, a lab strain) and differ from each other by ~2×10^4^ SNPs throughout the genome (49). Our environments varied by temperature (30°C and 37°C) and pH (3.2, 5.0 and 7.0), two stressors with global effects on yeast physiology (50–52), in a factorial design (see Methods, Figure S1). pH 3.2 and pH 7.0 are close to the lower and upper end of the viability range of our strains whereas both temperatures are well within the viability range. This choice of environments allowed us to explore a different (lower) range of growth rates of the background strains than in previous studies. Unlike previous studies, we kept our cultures growing close to the exponential steady state, which enabled us to infer the effect of each insertion mutation on absolute growth rate (GR, denoted by λ) from bulk barcode-based competition experiments, with precision of about 6.3×10^−^ h^−1^ (Methods).

We first estimated the effects of our mutations on GR in different background strains and environments. To this end, following Johnson et al (42), we designated a set of five mutations as a putatively neutral reference and found that the remaining 94 mutations exhibit a range of effects on GR relative to this reference, from decreasing it by Δλ ≈ 0.18 h^−1^ to increasing it by Δλ = 0.13 h^−1^, with the median effect Δλ ≈ 0 h^−1^. We validated a subset of these estimates with an independent low-throughput competition assay (Methods; Figure S2). We then classified each mutation in each strain and environment as either beneficial or deleterious if the 99% confidence interval around its estimated effect (Δλ) did not overlap zero (Methods). All other mutations were classified as neutral. This procedure yielded conservative calls of mutation sign, with a false discovery rate of 2.5%.

We found that the fraction of beneficial and deleterious mutations varied between 0% and 54% and between 0 and 62% per strain, respectively. However, no single mutation was identified as either beneficial, deleterious or neutral in all strains and environments. 94% (88/94) of our mutations are beneficial in at least one strain and condition, and, of those, 98% (86/88) are also deleterious in at least one strain and condition. Even within the same environment, between 33% (31/94) and 65% (61/94) of all mutations change sign across background strains, and between 10% and 39% of mutations change sign across environments in the same strain (Figure S3). Thus, the vast majority of mutations neither unconditionally increase nor unconditionally decrease GR across genetic backgrounds and environments.

**Figure 1.**
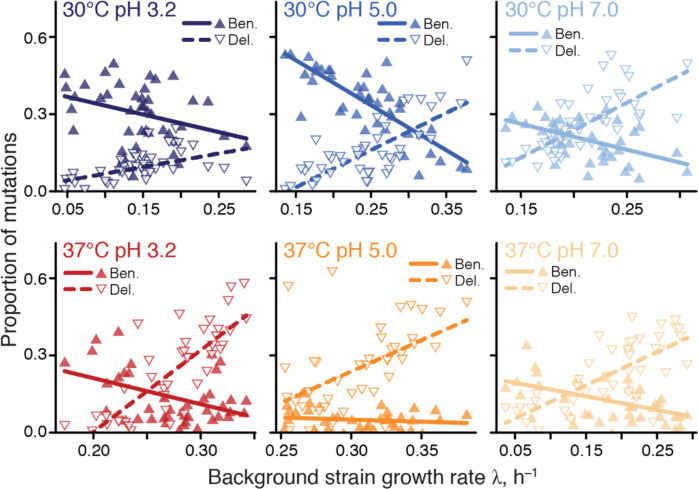
Proportions of beneficial and deleterious mutations vary with strain growth rate. Filled and empty triangles show the proportions of beneficial and deleterious mutations in each background strain as a function of its growth rate. Lines are the best-fit linear regressions; all are statistically significant (*P* < 0.05, t-test) except for beneficial mutations in 37°C pH 5.0.

A recent global epistasis model suggests that the effects of most mutations on GR should linearly decline with the GR of the background genotype (48). One striking qualitative prediction of this model is that the proportion of beneficial mutations should decline with the fitness of the background strain whereas the proportion of deleterious mutations should increase. While several previous studies found evidence for global epistasis (13, 37–46), none of them has observed sufficient numbers of mutational sign changes. Thus, we tested this qualitative prediction by looking for a correlation between strain GR and the proportion of mutations identified as beneficial and deleterious in that background. Consistent with the theory, we find that the proportion of beneficial and deleterious mutations decline and increase with background GR, respectively (Figure 1), and these relationships are statistically significant in all cases but one (beneficial mutations at 37°C pH 5). Thus, global epistasis is indeed a major determinant of the sign of mutations in all our environments (Figure S3C).

To probe the microscopic global epistasis model quantitatively, we modeled the effect $\Delta \lambda_{mge}$ of each mutation m on GR in strain g and environment e as

[1] $$\Delta \lambda_{mge}=a_{me}+b_{me}\lambda_{ge}+\xi_{mge},$$

where $\lambda_{ge}$ is the growth rate of the background genotype g and environment e. The first two terms in equation (1) capture global epistasis, a deterministic component which can be used for prediction, and $\xi_{ige}$ captures the remaining (unpredictable) epistasis, which we refer to as “idiosyncratic” (8, 39).

**Figure 2:**
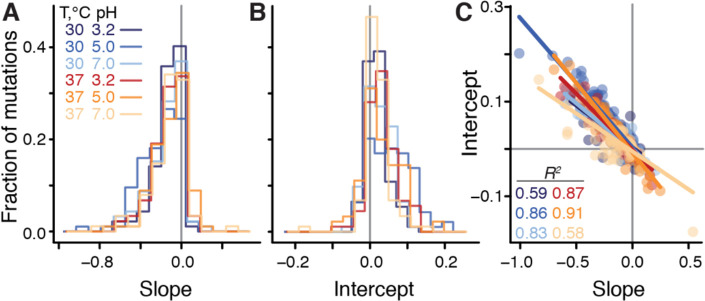
Distributions of global-epistasis slopes and intercepts and their correlation. **A.** Distributions of slopes from fitting Eq. (1) to data are largely indistinguishable between environments (see Figure S4 for statistical tests). **B.** Distributions of intercepts vary across environments (see Figure S4 for statistical tests). **C.** Correlation between slopes and intercepts. Each point represents a mutation, colored by environment. Lines are the best fit linear regressions (*P* < 0.01 for all, t-test).

We found that the linear model (1) was statistically significant for 94% (88/94) of mutations (*F*-test, *P* < 0.05 after Benjamini-Hochberg correction), and explained on average 46% (interquartile interval [35%, 60%]) of variance in the effects of mutations across background strains and environments (Methods). When tested individually, 38% (205/545) of global epistasis slopes $b_{ie}$ are significantly different from zero (t-test*, P* < 0.05 after Benjamini-Hochberg correction), with 98% (200/205) of them being negative (Figure 2A), consistent with the global epistasis theory (48) and previous observations (39, 42). 96% (196/205) of significant intercepts $a_{ie}$ are positive (Figure 2B), implying that most mutations are expected to increase GR in a hypothetical non-growing strain, which is consistent with the relationship between background GR and the proportions of beneficial and deleterious mutations observed in Figure 1.

We next sought to understand how the global epistasis slopes $b_{ie}$ and intercepts $a_{ie}$ vary across mutations and environments. We found that distributions of slopes are nearly invariant across environments (Figures 2A, S4A and Table S1), whereas the distributions of intercepts vary across environments (Figure 2B, S4A and Table S1). Furthermore, slopes and intercepts are strongly negatively correlated (Figure 2C, S4), such that mutations with a zero slope have on average a zero intercept, which explains the paucity of unconditionally deleterious and beneficial mutations noted above. This relationship further implies that the global epistasis intercepts can be expressed as $a_{me}=-{\overset{‾}{\lambda }}_{e}b_{me}+\eta_{me}$. Here, the environment-specific regression coefficient ${\overset{‾}{\lambda }}_{e}$ has a clear biological interpretation: it is the “pivot GR” at which a typical mutation switches its sign (Methods). The terms $\eta_{me}$, which we refer to as the “pivot noise”, can be modeled as normal random variables with zero mean and the same variance across all environments (Figure S4B).

**Figure 3.**
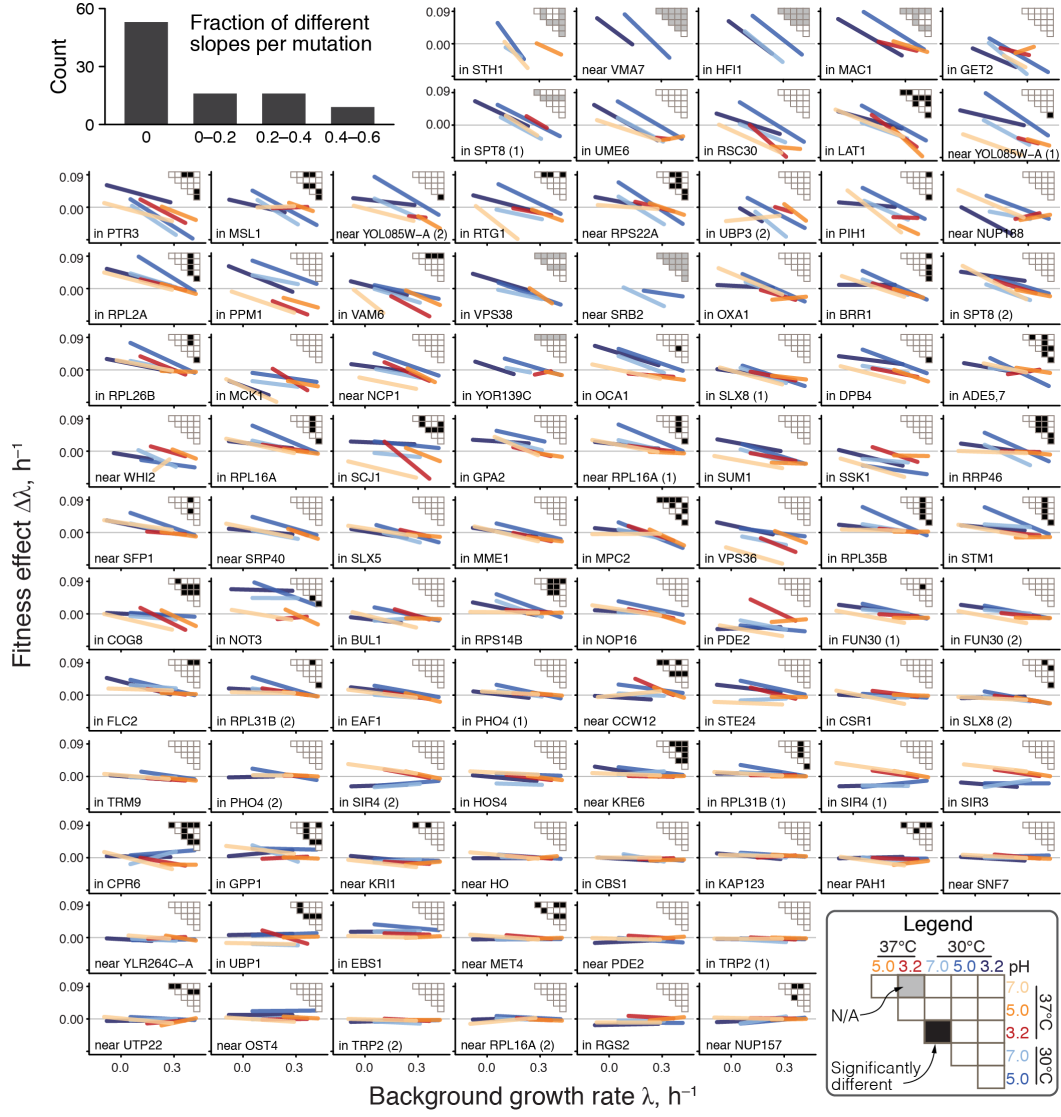
Global-epistasis slopes mutations are nearly invariant across environments. Panels show regression lines from fitting Eq. (1) for each mutation, colored by the environment as in previous figures. Mutations are displayed in the order of increasing mean slope. Insets show the results of all pairwise slope-comparison tests (legend in lower right). Histogram in top left shows the overall distribution of the fraction of significant test per mutation (see Methods for details).

We next sought to understand why the distributions of global epistasis slopes are nearly invariant across environments. We found that this near-invariance arises because of the near-invariance of slopes of individual mutations. Indeed, slopes of individual mutations are statistically indistinguishable across environments in 86% (1153/1333) of pairwise comparisons (Figures 3, S4, S5), and 56% (53/94) of mutations have statistically indistinguishable slopes in all environments. Moreover, even when slopes are statistically distinguishable, they are very similar, so that a model with six environment-specific slopes $b_{ie}$ explains only 4% more variance in the effects of mutations compared to an “invariant slope” model where each mutation is characterized by a single environment-independent slope $b_{m}$ (46% versus 42% on average). The near-invariance of global-epistasis slopes of individual mutations could arise trivially if each environment shifted the GRs of all strains by the same amount while preserving the relative order of their GRs and the effects of mutations. However, this is not the case. Slopes are nearly preserved despite the fact that the relative rank orders of background strains and mutations are reshuffled across environments (Figures S6, S7).

Taken together, the near-invariance of global-epistasis slopes across environments and the linear relationship between slopes and intercepts indicate that the microscopic G×G, G×E, and G×G×E interactions for most of our mutations are largely captured by a simplified version of equation (1),

[2] $$\Delta \lambda_{mge}=b_{m}\left(\lambda_{ge}-{\overset{‾}{\lambda }}_{e}\right)+\zeta_{mge},$$

where $\zeta_{mge}=\eta_{me}+\xi_{mge}$. Equation (2), which we refer to as the “generalized global epistasis equation”, shows that the effect of the environment on global epistasis is captured by a single effective parameter, the pivot growth rate ${\overset{‾}{\lambda }}_{e}$.

To understand the implications of equation (2) for the macroscopic G×G, G×E and G×G×E interactions, we calculated the first three moments of the distribution of fitness effects of mutations (DFE) under the simplifying assumption that pivot noise $\eta_{me}$ and idiosyncratic epistasis $\xi_{me}$ terms are all independent (see Methods for details). As expected, the generalized global epistasis equation predicts that the DFE mean ought to decline linearly with the background GR, such that this line has a slope invariant across environments and crosses zero at the environment-specific pivot GR ${\overset{‾}{\lambda }}_{e}$.

The behavior of higher moments of the DFE is less obvious. We find that the DFE variance is predicted to depend on $\lambda_{ge}$ quadratically, with the parabola’s minimum achieved at the pivot GR.

**Figure 4.**
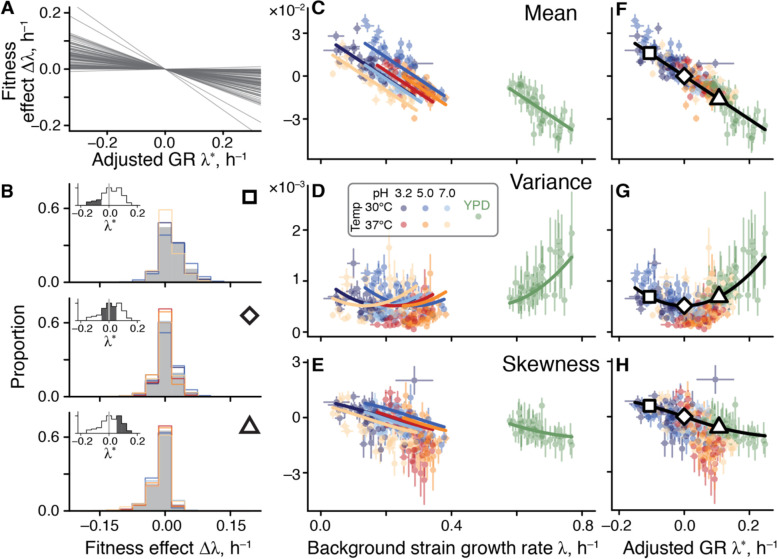
Generalized global epistasis equation explains the variation in the distribution of fitness effects across strains and environments. **A.** The effects of mutations in 30°C pH 7.0 according to the generalized global epistasis equation with the ζ-term set to zero. Each line represents a mutation. **B.** Estimated DFEs for strains whose adjusted GR is negative (top panel), approximately zero (middle panel) and positive (bottom panel). Gray bars show DFEs pooled across all environments, colored lines show DFEs for individual environments (colors are as in previous figures). Insets show the distributions of adjusted GRs for background strains, with the focal adjusted GR bin highlighted. Large square, rhombus and triangle are shown for reference with panels F,G,H. **C, D, E.** DFE moments plotted against the background strain GR. Error bars show ±1 standard errors (see Methods). Solid curves show the values calculated from Eq. (2) (Methods), and parameterized without the YPD data from Ref. (42). **F, G, H.** Same data as in C, D, E, but plotted against the adjusted GR. Theoretical curves explain 78%, 44%, and 33% of variation for the DFE mean, variance and skewness, respectively (*P* < 2.2×10^−16^).

To understand this prediction intuitively, consider an idealized case where the effects of all mutations vary according to the generalized global epistasis equation with $\zeta_{mge}$ set to zero (Figure 4A). Then, all mutations switch sign exactly at the pivot GR, so that a strain whose GR equals to ${\overset{‾}{\lambda }}_{e}$ has access only to neutral mutations. The DFE of such a strain has zero variance. Since the global epistasis lines for individual mutations spread out as the background GR deviates further from ${\overset{‾}{\lambda }}_{e}$ in either direction, the DFE variance increases. When $\zeta_{mge}\neq 0$, this overall pattern still holds but the variance at the pivot GR becomes positive (Figure S8).

Finally, the generalized global epistasis equation predicts that the skewness of the DFE ought to decline monotonically with $\lambda_{ge}$ and cross zero again at the pivot GR. More generally, our model makes a qualitative prediction that the DFE varies across strains as a function of their environment-adjusted GR $\lambda_{ge}^{*}=\lambda_{ge}-{\overset{‾}{\lambda }}_{e}$ rather than as a function of their absolute GR $\lambda_{ge}$. Furthermore, in all environments, all odd central moments of the DFE are expected to be zero when the adjusted GR equals zero and all even central moments of the DFE are expected to achieve their minimum at the same value (Methods).

To test these predictions, we compared the empirical DFEs across environments. We find that pairs of strains with matched adjusted GRs have significantly more similar DFEs than pairs of strains with the same absolute GR in different environments or the same strain in different environments (Figure 4B and S9), consistent with our predictions. We then plotted the first three moments of the empirical DFEs against the unadjusted and adjusted GR in all environments. We find that these moments align remarkably well when plotted against the adjusted GR but not when plotted against the absolute GR (Figures 4C–H). As predicted, the DFE mean and skewness decline monotonically with the strain’s adjusted growth rate and cross zero when the adjusted growth rate vanishes. As predicted, the DFE mean is a linear function whose slope is invariant across environments (Figure 4C,H). The most non-trivial prediction, that the DFE variance is a non-monotonic function of the adjusted GR whose minimum is achieved at zero adjusted GR, also holds.

The fact that all our predictions hold indicates that the linear generalized global epistasis equation with uncorrelated noise terms quantitatively captures the variation in the DFE shape across genotypes and environments. However, if the environment truly modulates only one effective parameter, the pivot GR, then we should be able to predict DFE shapes in any environment once its pivot GR is known. To test this prediction, we turned to our previous work where we measured DFEs of 163 yeast strains (a superset of the 42 strains used in this study) in a rich YPD environment (42). We estimated the pivot GR for this environment as the background GR where the mean of the DFE equals zero (see Methods for details). After GR adjustment, we found that our theoretical predictions quantitatively capture variation in the DFE moments across strains without any other fitted parameters (green points in Figure 4C–H).

These results show that microscopic global epistasis imposes simple, predictable, and general constraints on the G×G, G×E and G×G×E interactions at the macroscopic level. Specifically, the external environment controls a single effective parameter, the pivot growth rate ${\overset{‾}{\lambda }}_{e}$; strains that grow much slower than ${\overset{‾}{\lambda }}_{e}$, have wide, positively skewed DFEs with a positive mean; strains whose GR is close to ${\overset{‾}{\lambda }}_{e}$ have narrow, symmetric DFEs with a zero mean; and strains that grow much faster than ${\overset{‾}{\lambda }}_{e}$ have wide, negatively skewed DFEs with a negative mean (Figure 4B).

To the extent that our observations hold beyond the specific set of strains, mutations and environments investigated in this study, they have a number of important implications. In genetics, the generalized global epistasis model can be incorporated into QTL analyses to improve predictions of the phenotypic effects of mutations. In evolutionary biology, our results point to the existence of a universal class of distributions of fitness effects of mutations, which could explain why evolutionary dynamics of fitness are so similar and predictable across systems (30, 39, 46, 53–55). In conservation biology, the fact that low fitness genotypes have access to surprisingly large supplies of beneficial mutations gives hope that evolutionary rescue may prevent some species extinctions. More fundamentally, our results support the idea that epistasis effectively reduces the dimensionality of genotype-to-phenotype maps (48, 56, 57). These biological constraints that cause this remarkable dimensionality reduction are not well understood. Why they emerge and when they break down are exciting open questions in systems biology.

## Supplementary Material

Supplement 1

Supplement 2

Supplement 3

## Data Availability

Data described in the paper are presented in the supplementary materials. Raw sequencing data are publicly available at the NCBI Sequence Read Archive (accession no. PRJNA1028648), and all analysis code is available on GitHub (https://github.com/ardellsarah/Yeast_mutation_effects_across_strains_and_environments).
