# Environment-independent distribution of mutational effects emerges from microscopic epistasis

**DOI:** 10.1101/2023.11.18.567655

**Published:** 2024-05-13

**Authors:** Sarah Ardell, Alena Martsul, Milo S. Johnson, Sergey Kryazhimskiy

**Affiliations:** 1 Department of Ecology, Behavior and Evolution, University of California San Diego, La Jolla, CA 92093; 2 Department of Integrative Biology, University of California Berkeley, Berkeley, CA 94720; 3 Biological Systems and Engineering Division, Lawrence Berkeley National Laboratory, Berkeley, CA, USA.

## Abstract

Predicting how new mutations alter phenotypes is difficult because mutational effects vary across genotypes and environments. Recently discovered global epistasis, where the fitness effects of mutations scale with the fitness of the background genotype, can improve predictions, but how the environment modulates this scaling is unknown. We measured the fitness effects of ~100 insertion mutations in 42 strains of *Saccharomyces cerevisiae* in six laboratory environments and found that the global-epistasis scaling is nearly invariant across environments. Instead, the environment tunes one global parameter, the background fitness at which most mutations switch sign. As a consequence, the distribution of mutational effects is predictable across genotypes and environments. Our results suggest that the effective dimensionality of genotype-to-phenotype maps across environments is surprisingly low.

## One Sentence Summary

The effects of mutations on microbial growth rate follow a pattern of global epistasis that is invariant across environments.

Adaptive evolution can lead to profound changes in the phenotypes and behaviors of biological systems, sometimes with adverse and sometimes with beneficial consequences for human health, agriculture and industry (1–4). However, predicting these changes remains difficult (5–7). One major challenge is that how new mutations alter phenotypes and fitness of organisms often depends on the genetic background in which they arise (G×G interactions or “epistasis”), the environment (G×E interactions), or both (G×G×E interactions) (8). These interactions can alter not only the magnitude but also the sign of mutational effects, causing evolutionary trajectories to become contingent on the initial genotype, environment and random events (9).

Much of prior empirical work focused on characterizing “microscopic” G×G, G×E and G×G×E interactions for fitness-related phenotypes, that is, how the effects of individual mutations vary across genetic backgrounds and environments (10–19). Microscopic interactions determine evolutionary accessibility of mutational paths and the degree of genetic parallelism (8, 10, 20–22). However, using this approach for predicting genome evolution is challenging because the number of interactions grows super-exponentially with the number of variable loci (7, 23). Predicting phenotypic evolution may be more feasible and in many cases more useful (7, 24). Such predictions rely on coarse-grained, or “macroscopic”, descriptions of G×G, G×E and G×G×E interactions which ignore individual mutations and instead describe how the distributions of mutational effects vary across genotypes and environments (8, 20, 25, 26). While many studies measured the distributions of effects of mutations on fitness, or “DFEs” (27–33), a systematic understanding of macroscopic epistasis is still lacking (26, 31, 33, 34).

Several recent studies found that mutations often make the phenotype of an organism in which they occur less extreme (11, 12, 34–42), an instance of a more general phenomenon of microscopic “global epistasis” (8, 43). Such epistasis is expected to arise for complex traits, including fitness (44), and it can be used to quantitatively predict the effects of individual mutations in new genetic backgrounds without the full knowledge of their G×G interactions, thereby avoiding the combinatorial problem mentioned above (43, 44). Perhaps more importantly, if most mutations exhibit global epistasis, the distributions of their phenotypic effects should also have a quantitatively predictable shape (44), which could facilitate evolutionary predictions at the phenotypic level. However, we have limited understanding of how the patterns of microscopic global epistasis vary across environments (19), and no attempt has been made so far to empirically characterize how microscopic G×G, G×E and G×G×E interactions constrain the shape of the DFE.

Probing whether global epistasis models can capture G×G, G×E and G×G×E interactions at both microscopic and macroscopic levels hinges on measuring the effects of many mutations across multiple genetic backgrounds and environments. To this end, we measured how ~100 quasi-random barcoded insertion mutations constructed in our previous study (39) affect growth rate in 42 “background” strains of yeast *Saccharomyces cerevisiae* in six conditions (45). All background genotypes are segregants from a cross between two strains of yeast (RM, a vineyard strain, and BY, a lab strain) and differ from each other by ~2×10^4^ SNPs throughout the genome (46). Our environments varied by temperature (30°C and 37°C) and pH (3.2, 5.0 and 7.0), two stressors with global effects on yeast physiology (47–49), in a factorial design (see (45), Section 1.1 and Figure S1). This choice of strains and environments allowed us to explore a different (lower) range of growth rates of the background strains than in previous studies. Unlike previous studies, we kept our cultures growing close to the exponential steady state, which enabled us to infer the effect of each insertion mutation on absolute growth rate (denoted by λ) from bulk barcode-based competition experiments, with precision of about 7×10^−3^ h^−1^ (see (45), Section 1.3.3).

## Absence of unconditionally beneficial or deleterious mutations

We first estimated the effects of our mutations on growth rate in different background strains and environments. To this end, following Johnson et al (39), we designated a set of five mutations as a putatively neutral reference and found that the remaining 94 mutations exhibit a range of effects on growth rate relative to this reference (denoted by ∆λ), from decreasing it by ∆λ = −0.18 h^−1^ to increasing it by ∆λ = 0.14 h^−1^, with the median effect ∆λ ≈ 0 h^−1^. We validated a subset of these estimates with an independent low-throughput competition assay (see (45), Section 1.2.3, Figure S2C). We then classified each mutation in each strain and environment as either beneficial or deleterious if the 99% confidence interval around its estimated effect did not overlap zero (see (45), Section 1.3.4). All other mutations were classified as neutral. This procedure yielded conservative calls of mutation sign, with a false discovery rate of 2.4%.

We found that the fraction of beneficial and deleterious mutations varied between 0% and 63% and between 0 and 59% per strain, respectively. The high proportions of beneficial mutations was unexpected, but not unprecedented (30, 32). However, no single mutation was identified as either beneficial or deleterious in all strains and environments. 94% (88/94) of our mutations are beneficial in at least one strain and condition, and, of those, 96% (85/88) are also deleterious in at least one strain and condition. Even within the same environment, between 33% (31/94) and 63% (59/94) of all mutations change sign across background strains, and between 1% and 41% of mutations change sign across environments in the same strain (Figure S3). Thus, the vast majority of mutations neither unconditionally increase nor unconditionally decrease growth rate.

**Figure 1.**
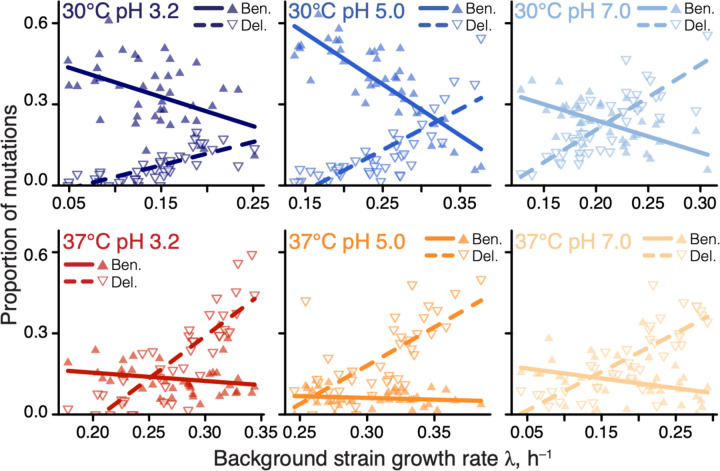
Proportions of beneficial and deleterious mutations vary with strain growth rate. Filled and empty triangles show the proportions of beneficial and deleterious mutations in each background strain as a function of its growth rate, respectively. Lines are the best-fit linear regressions; all are statistically significant (*P* < 0.05, t-test) except for beneficial mutations in 37°C pH 3.0 and 37°C pH 5.0.

A recent global epistasis model suggests that the fitness effects of most mutations should on average linearly decline with the fitness of the background genotype (44). One striking qualitative prediction of this model is that the proportion of beneficial mutations should increase with the decreasing fitness of the background strain, reaching up to 100% in the lowest-fitness genotypes, whereas the proportion of deleterious mutations should correspondingly decrease. Consistent with this prediction, we found that the proportion of beneficial and deleterious mutations increased and declined in slow-growing strains, respectively, and these relationships were statistically significant in 10/12 cases (Figure 1). Thus, global epistasis indeed appears to be a major determinant of the sign of mutations (Figure S3C).

## Consistency of microscopic global epistasis across environments

To probe the microscopic global epistasis model quantitatively, we modeled the effect $\text{Δ}\lambda_{mge}$ of each mutation m on growth rate in strain g and environment e as

[1] $$\text{Δ}\lambda_{mge}=a_{me}+b_{me}\lambda_{ge}+\xi_{mge},$$

where $\lambda_{ge}$ is the growth rate of the background genotype g in environment e. The first two terms in equation (1) capture global epistasis, a deterministic component which can be used for prediction, and $\xi_{mge}$ captures the remaining (unpredictable) epistasis, which we refer to as “idiosyncratic” (8, 36).

**Figure 2:**
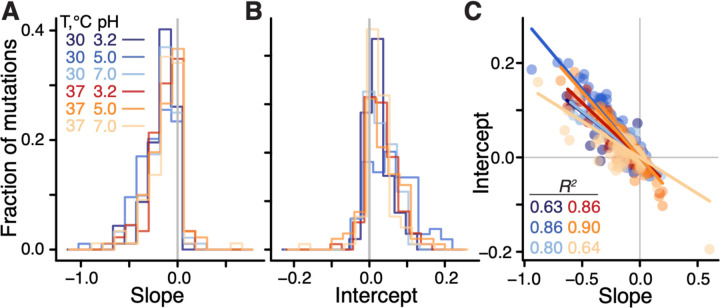
Distributions of global-epistasis slopes and intercepts and their correlation. **A, B.** Histograms of slopes and intercepts estimated from fitting equation (1) to data (see Figure S4 for statistical tests). **C.** Correlation between slopes and intercepts. Each point represents a mutation, colored by environment. Lines are the best fit linear regressions (*P* < 0.01 for all, t-test).

We found that the linear model (1) was statistically significant for 97% (91/94) of mutations (*F*-test, *P* < 0.05 after Benjamini-Hochberg correction), and explained on average 45% (interquartile interval [30%, 61%]) of variance in the effects of mutations across background strains and environments (see (45) Section 1.3.7). When tested individually, 46% (250/545) of global epistasis slopes $b_{me}$ are significantly different from zero (t-test*, P* < 0.05 after Benjamini-Hochberg correction), with 98% (245/250) of them being negative (Figure 2A), consistent with the global epistasis theory (44) and previous observations (36, 39). 96% (240/250) of significant intercepts $a_{me}$ are positive (Figure 2B), implying that these mutations are expected to be beneficial in a hypothetical non-growing strain, consistent with relationships shown in Figure 1.

We next examined how the global epistasis slopes $b_{me}$ and intercepts $a_{me}$ vary across mutations and environments. We found that the distributions of slopes are nearly invariant across environments (Figures 2A, S4A,E and Table S1), while the distributions of intercepts are more variable (Figure 2B, S4A and Table S1). Furthermore, slopes and intercepts are strongly negatively correlated (Figure 2C, S4F), such that mutations with a zero slope have on average a zero intercept, which is consistent with the paucity of unconditionally deleterious and beneficial mutations noted above. Thus, global epistasis slopes and intercepts are mathematically related as $a_{me}=-{\bar{\lambda }}_{e}b_{me}+\eta_{me}$ where the regression coefficient ${\bar{\lambda }}_{e}>0$ can be interpreted as the “pivot” growth rate at which a typical mutation switches its sign in environment e (see (45), Section 1.3.7). Each individual mutation switches its sign at a background growth rate that deviates from ${\bar{\lambda }}_{e}$ by $\eta_{me}/b_{me}$, and we therefore refer to $\eta_{me}$ as the “pivot noise”. We find that the distributions of $\eta_{me}$ in all environments are close to normal, with zero mean and standard deviation of approximately 1.7×10^−2^ (Figure S4C). On the other hand, pivot growth rates ${\bar{\lambda }}_{e}$ differ significantly across environments (see (45), Section 1.3.7; Figure S4 B,G).

**Figure 3.**
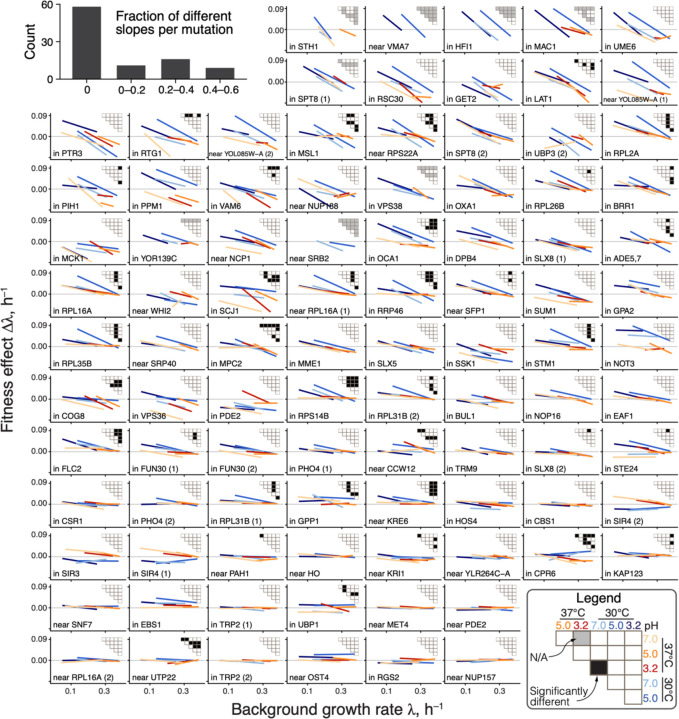
Global-epistasis slopes of mutations are nearly invariant across environments. Panels show regression lines from fitting equation (1) for each mutation, colored by the environment as in previous figures. Data points are shown in Figure S6. Mutations are displayed in the order of increasing mean slope. Insets show the results of all pairwise slope-comparison tests (legend in lower right). Histogram in top left shows the overall distribution of fractions of significant tests per mutation (see (45), Section 1.3.7).

There are two possibilities for how the distributions of global-epistasis slopes could be nearly invariant across environments. It could be because slopes of individual mutations are nearly invariant. Alternatively, slopes of individual mutations could change across environments, but their distribution is preserved. To test these hypotheses, we examined how the global-epistasis slope of each individual mutation changes across environments. We found that they are statistically indistinguishable in 87% (1169/1333) of pairwise comparisons, and 62% (58/94) of mutations have statistically indistinguishable slopes in all environments (Figures 3, S5, S6). Moreover, even when slopes are statistically distinguishable, they are very similar, so that a model with six environment-specific slopes $b_{me}$ explains only 2% more variance in the effects of mutations compared to an “invariant slope” model where each mutation is characterized by a single environment-independent slope $b_{m}$ (45% versus 43% on average; Figure S5). These results hold even if we only compare non-zero slopes or control for missing measurements (see (45), Section 1.3.7 and Figures S5).

The near-invariance of global-epistasis slopes of individual mutations could arise trivially if each environment shifted the growth rates of all strains by the same amount while preserving the relative order of their growth rates and the effects of mutations. However, this is not the case. We find that 34 out of 42 (81%) background strains are found among the 50% fastest growing strains in at least one environment and also among the 50% slowest growing strains in at least one other environment (see (45), Section 1.3.6 and Figure S7). Similarly, 50% of mutations on average switch between the top and bottom 50% of mutation effects in the same strain across environments (Figures S8). Thus, slopes are nearly preserved despite the fact that the relative rank orders of background strains and mutations are reshuffled across environments.

Taken together, the near-invariance of global-epistasis slopes across environments and the linear relationship between slopes and intercepts indicate that the microscopic G×G, G×E, and G×G×E interactions for most of our mutations follow a simplified version of equation (1),

[2] $$\text{Δ}\lambda_{mge}=b_{m}\left(\lambda_{ge}-{\bar{\lambda }}_{e}\right)+\eta_{me}+\xi_{mge}.$$

Equation (2), which we refer to as the “generalized global epistasis equation”, shows that the deterministic effect of the environment on global epistasis is captured by a single effective parameter, the pivot growth rate ${\bar{\lambda }}_{e}$.

## Distribution of fitness effects

To understand the implications of equation (2) for the macroscopic G×G, G×E and G×G×E interactions, we calculated the first three moments of the distribution of fitness effects of mutations (DFE) under the simplifying assumption that pivot noise $\eta_{me}$ and idiosyncratic epistasis $\xi_{mge}$ terms are all independent (see (45), Section 1.4). The generalized global epistasis equation predicts that the DFE mean ought to decline linearly with the background growth rate, with the same slope across environments, crossing zero at the environment-specific pivot growth rate ${\bar{\lambda }}_{e}$.

**Figure 4.**
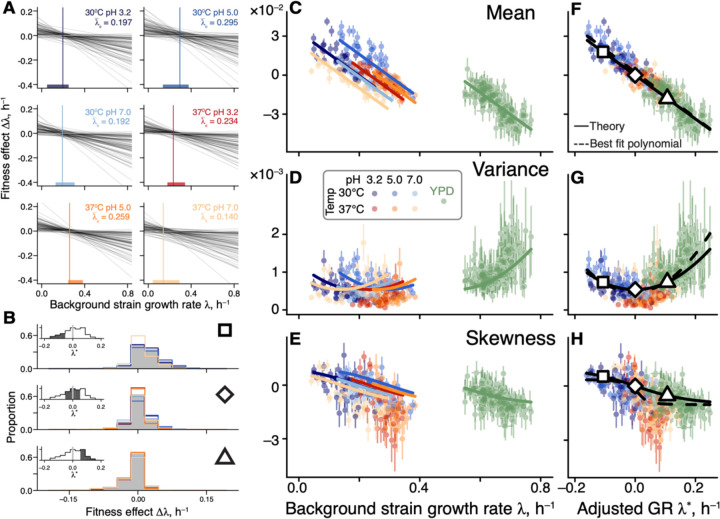
Generalized global epistasis equation captures changes in the DFE across strains and environments. **A.** The global epistasis contribution to the fitness effect of each mutation (term $b_{m}\left(\lambda_{ge}-{\bar{\lambda }}_{e}\right)$) in equation (2)) as a function of background growth rate term $\lambda_{ge}$. Each line represents a mutation. Vertical line indicates the pivot growth rate. Thick colored lines on the *x*-axis indicate the range of measured background-strain growth rates in each environment. **B.** Estimated DFEs for strains whose adjusted growth rate is negative (top panel), approximately zero (middle panel) and positive (bottom panel). Gray bars show DFEs pooled across all environments, colored lines show DFEs for individual environments (colors are as in previous figures). Insets show the distributions of adjusted growth rates for background strains, with the focal bin shaded. Large square, rhombus and triangle are shown for reference with panels F,G,H. **C, D, E.** DFE moments plotted against the background strain growth rate. Error bars show ±1 standard errors (see (45), Section 1.3.9). Solid curves show the theoretical predictions calculated from equation (2) and parameterized without the YPD data (see (45), Section 1.4). DFE moments are calculated with a median of 74 mutations (interquartile interval [61,79]). **F, G, H.** Same data as in C, D, E, but plotted against the adjusted growth rate. Dashed curves are the best fitting polynomial of the corresponding degree (see (45), Section 1.4).

The behavior of higher DFE moments is less obvious. DFE variance is predicted to depend on $\lambda_{ge}$ quadratically, with the parabola’s minimum achieved at the pivot growth rate. To understand this prediction intuitively, consider an idealized case without idiosyncratic epistasis $\xi_{mge}=0$ (Figure 4A). Since most mutations switch sign near the pivot growth rate, strains growing at this rate have access only to mutations with small effects, and the DFE is narrow. As the background growth rate deviates from ${\bar{\lambda }}_{e}$, global epistasis lines for individual mutations spread out forming a “bowtie” (Figure 4A), and the DFE variance increases in both directions. This general pattern still holds even when $\xi_{age}\neq 0$ (see (45), Section 1.4).

Finally, DFE skewness is predicted to decline monotonically with $\lambda_{ge}$, crossing zero again at the pivot growth rate. More generally, our model makes a qualitative prediction that the DFE varies across strains as a function of their environment-adjusted growth rate $\lambda_{ge}^{*}=\lambda_{ge}-{\bar{\lambda }}_{e}$ rather than as a function of their absolute growth rate $\lambda_{ge}$. Furthermore, when the adjusted growth rate is zero, all odd central moments of the DFE are predicted to vanish and all even central moments are predicted to achieve their minimum (see (45), Section 1.4).

To test these predictions, we compared the empirical DFEs across environments. We find that pairs of strains with matched adjusted growth rates have significantly more similar DFEs than pairs of strains with the same absolute growth rate in different environments or the same strain in different environments (Figure 4B and S9), consistent with our predictions. We then plotted the first three moments of the empirical DFEs against the unadjusted and adjusted growth rate in all environments. We find that these moments align well only in the latter plot (Figures 4C–H). As predicted, the DFE mean and skewness decline monotonically with the strain’s adjusted growth rate $\lambda_{ge}^{*}$ and cross zero when $\lambda_{ge}^{*}=0$, and DFE variance achieves its minimum at $\lambda_{ge}^{*}=0$. We confirmed that these patterns are not a result of missing measurements (see (45), Section 1.3.9 and Figure S10).

The fact that all of our predictions hold indicates that the linear generalized global epistasis equation with uncorrelated noise terms quantitatively captures a major mode of variation in the DFE shape across genotypes and environments. However, if the environment truly modulates only one effective parameter, the pivot growth rate, then we should be able to predict DFE shapes in any environment, once its pivot growth rate is known. To test this prediction, we turned to our previous work where we measured DFEs of 163 yeast strains (a superset of the 42 strains used in this study) in a rich medium YPD (39). We estimated the pivot growth rate for this environment as the background growth rate where the DFE mean equals zero (see (45), Section 1.3.5). After growth-rate adjustment, we found that our theoretical predictions quantitatively capture variation in the DFE variance and skewness without any other fitted parameters (green points in Figure 4C–H).

## Discussion

Having measured the fitness effects of many mutations across diverse yeast genotypes and environments, we obtained two main results. First, the global-epistasis slopes of individual mutations are nearly invariant across environments. If interpreted through the lens of the Reddy-Desai model (44), this finding suggests that our environmental perturbations largely preserve the overall statistical structure of genetic interactions, consistent with previous work in yeast (50). Whether this regularity holds for more extreme perturbations or those that target specific cellular processes, such as drugs, remains to be seen (19). The near-invariance of global-epistasis slopes across environments leads to a simple equation (2) that allows us to predict the expected fitness effect of a mutation in a new strain and environment. However, it is important to keep in mind that the actual effect will deviate from this expectation due to idiosyncratic epistasis. In other words, equation (2) is statistical in nature and, as such, does not uniquely determine the underlying genotype-to-fitness map (see (45), Section 2.3).

Our second main result is the empirical characterization of constraints imposed on the DFE by microscopic global epistasis. We found that the environment controls a single effective parameter, the pivot growth rate ${\bar{\lambda }}_{e}$, and a genotype’s DFE is set by the deviation of its growth rate from ${\bar{\lambda }}_{e}$, as shown in Figure 4. For example, many of our background strains happen to grow slower than ${\bar{\lambda }}_{e}$ and, as a result, have access to many beneficial mutations (Figure 1).

The physiological mechanisms that determine the pivot growth rate are as of yet unclear. According to the Reddy-Desai model, “bowtie” patterns analogous to those shown in Figure 4A are expected to arise generically on genotype-to-fitness maps with many epistatic interactions (44). In this model, a typical mutation switches sign at the average fitness of all genotypes, indicating that the pivot growth rate could be a metric of environmental permissibility. The fact that in our data the pivot growth rate correlates with the average growth rate of our background strains is consistent with this interpretation (Figure S4G).

While the consistency of our results with theory suggests that they should extend to other strains and organisms, types of mutations and environments, their generality must ultimately be established empirically. If they hold broadly, several important implications are conceivable. In genetics, our model can improve predictions of the phenotypic effects of mutations. In conservation biology, the possibility that low-fitness genotypes have access to large supplies of beneficial mutations gives hope that evolutionary rescue may prevent some species extinctions. In evolutionary biology, our results point to the existence of a universal class of distributions of fitness effects of mutations, which could explain why evolutionary dynamics of fitness are so similar and predictable across systems (25, 36, 42, 51). More broadly, our model can help us better understand these dynamics, including how adaptation to one environment alters fitness in another (26) or how the DFE might change along an evolutionary trajectory (45). More fundamentally, our results suggest that epistasis reduces the effective dimensionality of genotype-to-phenotype maps (8, 43, 44, 52). What biological constraints cause this dimensionality reduction, how it emerges and when it breaks down are exciting open questions in systems biology.

## Supplementary Material

Supplement 1

Supplement 2

Supplement 3

## Data Availability

Data described in the paper are presented in the supplementary materials (Tables S1 through S6). Raw sequencing data are publicly available at the NCBI Sequence Read Archive (accession no. PRJNA1028648), and all analysis code is available on GitHub (53). Data for the YPD environment provided in Ref. (39).
