# Supplementary material for: Environment-independent distribution of mutational effects emerges from microscopic epistasis
Source: bioRxiv. 2023 Nov 18:2023.11.18.567655. Preprint. [Version 1] doi: 10.1101/2023.11.18.567655 (PMC10680819; doi:10.1101/2023.11.18.567655)
Supplement: Supplementary file 1 [file NIHPP2023.11.18.567655v1-supplement-3.pdf]

bioRxiv preprint doi: https://doi.org/10.1101/2023.11.18.567655; this version posted November 18, 2023. The copyright holder for this preprint (which was not certified by peer review) is the author/funder, who has granted bioRxiv a license to display the preprint in perpetuity. It is made available under aCC-BY 4.0 International license.

Supplementary Materials for

Environment-Independent Distribution of Mutational Effects Emerges from Microscopic Epistasis

Sarah Ardell, Alena Martsul, Milo S. Johnson, Sergey Kryazhimskiy*

*E-mail: skryazhi@ucsd.edu

This PDF file includes:

1 Materials and methods **2**
1.1 Strains and media 2
1.1.1 Background strains 2
1.1.2 RB-TnSeq libraries 2
1.1.3 Media and environments 2
1.2 Experimental procedures 3
1.2.1 RB-TnSeq experiment 3
1.2.2 Sequencing library preparation 3
1.2.3 Validation of estimated fitness effect of mutations 5
1.3 Data Analysis 6
1.3.1 Code 6
1.3.2 Counting barcodes 6
1.3.3 Estimating growth rates of background strains and fitness effects of mutations 6
1.3.4 Calling beneficial and deleterious mutations 10
1.3.5 Growth rates of background strains in YPD 10
1.3.6 Variation of growth rates and mutational effects across environments 10
1.3.7 Models of global epistasis 11
1.3.8 Variance partitioning for the sign of mutations 12
1.3.9 Empirical DFEs 14
1.3.10 QTL analysis 15
1.4 Theoretical calculation of DFE moments 16
2 Supplementary tables **20**
3 Supplementary figures **22**

bioRxiv preprint doi: https://doi.org/10.1101/2023.11.18.567655; this version posted November 18, 2023. The copyright holder for this preprint (which was not certified by peer review) is the author/funder, who has granted bioRxiv a license to display the preprint in perpetuity. It is made available under aCC-BY 4.0 International license.

1 Materials and methods

1.1 Strains and media

1.1.1 Background strains

Our "background strains" of yeast *S. cerevisiae* are a subset of a larger library of segregants that were previously generated from a cross between the lab strain BY and the vineyard strain RM (*1*) and whose evolutionary properties have been previously characterized (*2, 3, 4*). Specifically, our set of 42 background strains (listed in Table S4-Tab 2) is a subset of the strains used in the "Small Library" RB-TnSeq experiment described in Ref. (*3*). All the necessary strain details can be found in Refs. (*1, 3*). Most importantly, our background strains differ from each other at approximately 25,000 loci and span nearly the full growth rate range in YPD measured in Ref. (*3*) (see Section 1.3.5). These strains also vary widely in both DFE mean (2.5-fold range) and variance (1.5-fold range) in YPD.

1.1.2 RB-TnSeq libraries

Background strains individually transformed with these RB-TnSeq libraries were kindly provided by Michael Desai (Harvard University). The design and construction of the RB-TnSeq libraries is described in detail in Ref. (*3*). Briefly, each background strain was transformed twice with the same set of 100 redundently barcoded transposon-insertion mutations, resulting in two biological replicates for each mutation in each strain, such that, within each transformation, each barcode uniquely tags a particular mutation and background strain (43% of barcodes were used in both transformations). The list of mutations and their corresponding barcodes is provided in Table S4-Tab 4. On average, each mutation was represented by 11 and 37 barcodes in the first and second transformation, respectively (see Table S4-Tab 4). Five mutations (IDs: 91 (nearby COA6), 51 (nearby FIT2), 6 (nearby MET2), 99 (nearby TDA11), 102 (nearby YSP2); see Table S4-Tab 4) that target intergenic regions were used as a neutral reference, as in Ref. (*3*). These reference mutations were represented by on an average 19 and 77 in barcodes in the two transformations.

1.1.3 Media and environments

Unless otherwise noted, all experiments were performed in synthetic complete medium (SC, 2% dextrose (VWR, #90000-904), 0.67% YNB + nitrogen powder (Sunrise Science Products, #1501-500), 0.2% synthetic complete drop-out powder mixture (Sunrise Science Products,#1300-030)). We added ampicillin (Amp) and tetracycline (Tet) at concentrations given in Table S2 into the medium to prevent bacterial contamination. Our environmental conditions differed by two factors, temperature (30°C and 37°C) and pH (3.2, 5.0, and 7.0). pH was maintained with the citrate-phosphate buffer (*5*), which was prepared using 1 M stocks of citric acid (VWR, # 97061-858) and K_2HPO_4 (VWR, #97062-234) following the protocol described in Ref. (*6*). Autoclaved media were pH-adjusted by adding the necessary volumes of sterile citric acid and K_2HPO_4 solutions and measuring the pH of the buffered media. If the pH of the buffered media deviated from the desired level, we further adjusted it by adding small volumes of 4 M HCl (Sigma-Aldrich #84435). Media was used within two days.

bioRxiv preprint doi: https://doi.org/10.1101/2023.11.18.567655; this version posted November 18, 2023. The copyright holder for this preprint (which was not certified by peer review) is the author/funder, who has granted bioRxiv a license to display the preprint in perpetuity. It is made available under aCC-BY 4.0 International license.

1.2 Experimental procedures

1.2.1 RB-TnSeq experiment

To estimate the effects of tn-mutations on the absolute growth rate (GR) in 42 background strains in 6 environments, we pooled the tn-mutant libraries of our background strains into multiple pools (see below for details) and maintained these pools in continuous growth in 150 ml of media in 500 ml flasks over the period of 48 hours with dilutions down to 5×10^7 cells and sampling every 12 hours. We carried out two replicate competition assays, one per independently transformed library (see Section 1.1.2).

Pre-growth and pooling. Prior to pooling, mutagenized strain libraries were defrosted and pre-grown for 24 h in 96-deep-well plates with 1 ml of media in each of our environments. Based on preliminary GR estimates, we grouped the background-strain libraries by their GR into three groups for the competition assays at 30°C and into two groups for the competition assays at 37°C. Each background strain was represented only in one group per temperature, with the exception of LK5-G01, which was added to each group as cross-group control. Group identities of each background strain can be found in Table S4-Tab 2. Thus, we propagated 6 cultures (3 GR groups × 2 biological replicates) in each of the 30°C environments and 4 cultures (2 GR groups × 2 biological replicates) in each of the 37°C environments for a total of 30 cultures.

After pre-growth, we measured OD600 of each mutant culture and converted it into cell density using a previously obtained calibration curve. Based on these density estimates, we pooled mutant cultures at approximately equal abundances, with a slight over-representation of those background strains whose preliminary GR estimates were lower. We then measured the density of each mixed culture again and transferred 5×10^7 cells into the corresponding competition flask. The remaining mixed T_0 cultures were frozen using the protocol described below.

Growth and dilution. Competitions were carried out in 150 ml of media in 500 ml baffled flasks (Pyrex No. 4446-500) in a shaking incubator (Eppendorf New Brunswick I26, 2.5 cm orbit) set to 150 rpm and appropriate temperature. Every 12 h, we estimated the cell density of each culture using plate-reader-based OD600 measurements and a previously obtained calibration curve (Table S4-Tab 3). Then, 5×10^7 cells were transferred into the fresh media. When the transfer volume exceeded 1 ml, we adjusted the volume of fresh media to maintain the consistent culture volume of 150 ml. All cultures were propagated for four growth and dilution cycles (48 hours), yielding five samples per culture.

Sampling and storage. We pelleted the cells from 50 ml of each cultures remaining after the transfer and froze the cell pellets at −70°C for subsequent DNA extraction and barcode sequencing.

1.2.2 Sequencing library preparation

We used the YeaStar Genomic Kit Protocol I (Zymo Research, #D2002) to extract gDNA from ~ 1 ml of pelleted yeast cultures. To generate Illumina-ready dual-indexed amplicon library, we

bioRxiv preprint doi: https://doi.org/10.1101/2023.11.18.567655; this version posted November 18, 2023. The copyright holder for this preprint (which was not certified by peer review) is the author/funder, who has granted bioRxiv a license to display the preprint in perpetuity. It is made available under aCC-BY 4.0 International license.

used a two-step PCR protocol, modified from Ref. (7) as follows. All primer sequences can be found in Table S3.

First PCR. We combined 100 ng of extracted gDNA, 25 µl of OneTaq DNA polymerase Master Mix (New England BioLabs, #M0482L), 0.5 µl of 10 µM oAM-R2P-100-R01 primer, 0.5 µl of 10 µM oAM-R1P-20X-F01 primer, 1 µl of 50 µM $MgCl_2$, and molecular biology grade water up to the total volume of 50 µl. We used the following PCR protocol:

1. 94°C for 30 sec.
2. 94°C for 30 sec.
3. 50.5°C for 30 sec.
4. 68°C for 70 sec.
5. Repeat Steps 2–4 for a total of three times
6. 68°C for 5 min.

We purified this PCR product with AMPure XP magnetic beads (Beckman, #A36881) (1:1 ratio).

Second PCR. We combined 15 µl of purified PCR I product, 25 µl of OneTaq DNA polymerase Master Mix, 1 µl of 50 µM MgCl2, 1 µl of 10 µM N7XX primer (Nextera), and 1 µl of 10 µM S5XX primer (Nextera), and 7 µl of molecular biology grade H2O. We used the following PCR protocol:

1. 94°C for 30 sec.
2. 94°C for 30 sec.
3. 62°C for 30 sec.
4. 68°C for 70 sec.
5. Repeat Steps 2–4 for a total of 24 times
6. 68°C for 5 min.

The final PCR product was purified as above, run on a gel, extracted and purified with QIAquick PCR purification kit (Qiagen, #28106).

Sequencing. We sequenced the libraries with paired-end 150 bp reads on one HiSeq4000 platform and two HiSeq X10 platforms (Illumina).

bioRxiv preprint doi: https://doi.org/10.1101/2023.11.18.567655; this version posted November 18, 2023. The copyright holder for this preprint (which was not certified by peer review) is the author/funder, who has granted bioRxiv a license to display the preprint in perpetuity. It is made available under aCC-BY 4.0 International license.

1.2.3 Validation of estimated fitness effect of mutations

We were surprised by the high fraction of beneficial mutations identified in our RB-TnSeq experiment and carried out additional experiments designed to validate our fitness-effect estimates. To this end, after preliminary analyses, we selected a set of seven mutations: nearby MET2, nearby MET4, in NOT3, in PPM1, in RSC30, nearby TDA11, in MPC2 (Mutation IDs: 6,10, 117, 66, 71, 99, and 127, Table S4-Tab 4). We generated new RB-TnSeq libraries for these mutations and reconstructed them in two background strains, LK2-D07 and LK6-A05. Two of the selected mutations (nearby MET2 and nearby TDA11, IDs: 6, 99) were used as a neutral reference in the RB-TnSeq experiment. The mutation nearby MET4 (ID: 10) was identified to be neutral in the vast majority (74%) of genetic backgrounds and environments in the main experiment, and in all instances re-tested in the validation experiment. The remaining mutations were identified as more often beneficial in 30°C environments than 37°C and more often deleterious or neutral at 37°C than in 30°C. However, after final analyses, mutation 117 (in NOT3) no longer passed filters in the two focal strains and was excluded from the analyses.

The barcoded libraries for individual mutations were generated using the protocol described in Ref. (*3*). In total, we created 14 barcoded plasmid libraries (two replicated libraries per mutation), such that each library contained a unique set of barcodes. We then transformed these libraries into two background strains, LK2-D07 and LK6-A05, again following protocols described in Ref. (*3*). Transformant colonies were scraped and, after 24 h of additional growth in selective media, cultures were pelleted and frozen in 20% glycerol at -70°C. To determine which barcodes were associated with each mutation in each background strain, we sequenced each of the 14 yeast mutant libraries at the barcode locus using the same protocols as in Section 1.2.1. Barcode-mutation associations are provided in Table S4-Tab 8.

After generating the libraries of individual mutations, we estimated their fitness effects in two environments, 30°C pH 5.0 and 37°C pH 7.0, in each genetic background separately. We pre-grew 56 cultures (2 background strains $\times$ 7 mutations $\times$ 2 replicate mutant libraries $\times$ 2 environments) in the buffered SC media containing Amp (to avoid bacterial contamination) and either Nat or Hyg (required for selecting for the transformants, see Tables S4-Tab 2 and S2, and Ref. (*3*)) and incubated them in the two focal environments (30°C pH 5.0 and 37°C pH 7.0) for 24 h in test tubes shaken at 220 rpm. After measuring the concentrations of the grown cultures as described above (see Section 1.2.1), we created one mixed culture per background strain, per mutant library and per environment (2 environments $\times$ 2 strains $\times$ two biological replicates, for a total of 8 mixed cultures) as follows. We added each of the two neutral reference mutation cultures (IDs: 6,99) at frequency 25% each and we added each of the "query" mutant cultures (IDs: 10, 117, 66, 71, and 127) at 10%, such that initial ratio of reference and query mutants in each culture as 1:1. After estimating cell counts in these mixed cultures using OD600 (see Section 1.2.1), we transferred 5×10^7 cells to start the competition assay. The assays, sampling and sequencing library preparation were performed following the same protocols as in Section 1.2.1. Fitness effects of mutations estimated in this experiment are provide in Table S4-Tab 9.

bioRxiv preprint doi: https://doi.org/10.1101/2023.11.18.567655; this version posted November 18, 2023. The copyright holder for this preprint (which was not certified by peer review) is the author/funder, who has granted bioRxiv a license to display the preprint in perpetuity. It is made available under aCC-BY 4.0 International license.

1.3 Data Analysis

1.3.1 Code

All analysis code is written in R 4.3.1 and is available at

https://github.com/ardellsarah/Yeast_mutation_effects_across_strains_and_environments.

Packages used are listed in the beginning of all scripts. Computationally intensive analyses were run on the Triton Supercomputing Cluster (TSCC).

1.3.2 Counting barcodes

Raw barcode counts. Barcode counts for the main RB-TnSeq experiment (Section 1.2.1) were obtained using the BarcodeCounter2 package (*8*) with a pre-determined barcode-mutation association data from Ref. (*3*).

To determine barcode counts in the validation experiment, we first used the barcode sequencing data for individual mutation libraries (see Section 1.2.3) to associate each barcode sequence with a particular mutation and background strain (Table S4-Tab 4). To do so, we used regular expressions to extract all unique barcode sequences and clustered them using the `seq_cluster` function in R's bioseq package. We then used BarcodeCounter2 with the resulting barcode-mutation associations to extract raw barcode counts for each sample file.

Filtering. Because it is critical to have accurate reference barcode counts for the inference of fitness effects of mutations, we discarded all time points that contained less than 500 reference mutation counts for any given strain, replicate and environment. This filtering removed 1.3% (41,404/3,060,514) of strain-environment-replicate-time point combinations. Then, we retained only those barcodes that were present at three or more time points (in any given condition and replicate) at 5 or more counts at each time point.

1.3.3 Estimating growth rates of background strains and fitness effects of mutations

The central piece of our procedure for estimating the GRs of background strains and the fitness effects of mutations is the detection of "outlier" barcodes, i.e., those that have abnormally high or low GRs relative to other barcodes tagging the same mutation. Such outliers arise likely due to the tn-mutants acquiring secondary mutations either during the barcoding step or during the RB-TnSeq experiment. The outlier detection procedure requires preliminary estimates of fitness effects of barcodes tagging each mutation, which in turn requires a robust set of reference barcodes. Thus, our procedure consists of the following steps.

1. Estimate the GRs of all barcodes.
2. Detect and exclude outlier barcodes for reference mutations.
3. Obtain preliminary estimates of fitness effects of mutations based on the robust set of reference barcodes.

bioRxiv preprint doi: https://doi.org/10.1101/2023.11.18.567655; this version posted November 18, 2023. The copyright holder for this preprint (which was not certified by peer review) is the author/funder, who has granted bioRxiv a license to display the preprint in perpetuity. It is made available under aCC-BY 4.0 International license.

4. Detect and exclude outlier barcodes for non-reference mutations.
5. Estimate the background growth rates and the fitness effects of mutations in each biological replicate.
6. Pool estimates across replicates.

We describe the outlier detection algorithm at the end of this section. Suffices to say that this algorithm takes as input (i) the set of all barcodes tagging a given mutation (in a given genetic background, biological replicate and environment), (ii) the cell count estimates over time of all these barcoded lineages and (iii) the preliminary estimates of the fitness effects of these lineages, and it outputs a robust "outlier-free" set of barcodes corresponding to the mutation.

Before we describe our estimation procedure, recall that, in a given biological replicate r, each barcode k uniquely specifies a particular tn-mutation m and the background strain g into which this mutation is introduced (see Section 1.1.2). We denote the set of all barcodes tagging mutation m in genetic background g in replicate r by $\tilde{S}_{mgr}$. We also denote the set of five reference mutations by $\mathcal{S}^{\text{ref}}$ (see Section 1.1.2 and Table S4-Tab 4) and we denote the set of "reference barcodes", i.e., all barcodes that tag these reference mutations in the genetic background g and replicate r, by $\tilde{S}^{\text{ref}}_{gr} = \bigcup_{m\in\mathcal{S}^{\text{ref}}} \tilde{S}_{mgr}$. Tilde denotes the fact that these sets potentially include outlier barcodes.

Estimation of barcode GRs across time intervals. We first calculate the frequency of each barcode k (reference or non-reference) in each replicate r at each time point t by dividing its read count by the total count of all barcodes present in the same flask at that time. To estimate the number of cells N_{kret} that carry barcode k in repliate r in environment e at the sampling time t, we multiply barcode frequency by the total number of cells present in the flask at that time point (Table S4-Tab 3). We estimate the GR λ_{kret} of the barcode lineage k as

$$\lambda_{kret} = \frac{1}{\Delta t} \ln \frac{N_{kret+1}}{N_{kret}},$$

where Δt is the time between transfers (typically 12 h, Table S4-Tab 3).

Detection and exclusion of outlier reference barcodes. We obtain a preliminary estimate of the effect of each reference barcode k in background g, environment e and replicate r as

$$\widetilde{\Delta\lambda}_{kre} = \frac{1}{M_{kre}} \sum_t \left(\lambda_{kret} - \tilde{\lambda}_{gret}\right), \quad k \in \tilde{S}^{\text{ref}}_{gr},$$

where $\tilde{\lambda}_{gret} = \text{Median}\left\{\lambda_{kret} : k \in \tilde{S}^{\text{ref}}_{gr}\right\}$ and M_{kre} is the number of time intervals where barcode k is observed in environment e in replicate r. We use these estimates to apply our outlier detection algorithm (see below) and generate a robust set of reference barcodes S^{ref}_{gre} for each background strain g in each environment e and biological replicate r.

bioRxiv preprint doi: https://doi.org/10.1101/2023.11.18.567655; this version posted November 18, 2023. The copyright holder for this preprint (which was not certified by peer review) is the author/funder, who has granted bioRxiv a license to display the preprint in perpetuity. It is made available under aCC-BY 4.0 International license.

Preliminary estimates of fitness effects of mutations. For any barcode k tagging a non-reference mutation m in genotype g, we calculate its fitness effect in environment e and replicate r as

$$\Delta\lambda_{kre} = \frac{1}{M_{kre}} \sum_t \Delta\lambda_{kret}, \quad \text{for any } k \in \tilde{S}_{mgr}, \tag{S1}$$

where M_{kre} is the number of time intervals when this barcode is observed in environment e in replicate r, and

$$\begin{aligned} \Delta\lambda_{kret} &= \lambda_{kret} - \lambda_{gret}, \quad \text{for any } k \in \tilde{S}_{mgr}, \\ \lambda_{gret} &= \text{Median}\left\{\lambda_{kret} : k \in S^{\text{ref}}_{gre}\right\} \end{aligned} \tag{S2}$$

are robust estimates of the fitness effect of barcode k and GR of the background strain g, respectively, both at time interval t in environment e and replicate r. We then obtain a preliminary estimate of the fitness effect of each mutation m in genetic background g in environment e and biological replicate r as

$$\widetilde{\Delta\lambda}_{mgre} = \text{Median}\left\{\Delta\lambda_{kre} : k \in \tilde{S}_{mgr}\right\}. \tag{S3}$$

Detection and exclusion of outlier barcodes for non-reference mutations. We use preliminary fitness effect estimates given by equation (S3) and apply our outlier detection algorithm (see below) to generate a clean set of barcodes S_{mgre} for each mutation m in each background strain g in each environment e and biological replicate r.

Estimation of background GRs and fitness effects of mutations in each biological replicate. We estimate the GR of each background strain g in each environment e and biological replicate r as

$$\lambda_{gre} = \frac{1}{M_{gre}} \sum_t \sum_{k \in S^{\text{ref}}_{gre}} \lambda_{kret}, \tag{S4}$$

where M_{gre} is the number of barcode-time interval combinations at which λ_{kret} are estimated.

We estimate the fitness effect of each mutation m in each genetic background g environment e and biological replicate r as

$$\Delta\lambda_{mgre} = \frac{1}{M_{mgre}} \sum_t \sum_{k \in S_{mgr}} \Delta\lambda_{kret}, \tag{S5}$$

where $\Delta\lambda_{kret}$ are given by equation (S2) and M_{mgre} is the number of barcode-time interval combinations at which λ_{kret} are estimated.

Pooling estimates across biological replicates. We estimate how the fitness effects of mutations obtained using equation (S5) correlate across replicates. Since the replicates are highly correlated (Figure S2A-B), we pool the data from both replicates to obtain our final

bioRxiv preprint doi: https://doi.org/10.1101/2023.11.18.567655; this version posted November 18, 2023. The copyright holder for this preprint (which was not certified by peer review) is the author/funder, who has granted bioRxiv a license to display the preprint in perpetuity. It is made available under aCC-BY 4.0 International license.

estimates of background GRs and their standard errors,

$$\lambda_{ge} = \frac{1}{M_{ge}} \sum_{r,t} \sum_{k \in S^{\text{ref}}_{gre}} \lambda_{kret}, \tag{S6}$$

$$\sigma^{\lambda}_{ge} = \left[\frac{1}{M_{ge}(M_{ge}-1)} \sum_{r,t} \sum_{k \in S^{\text{ref}}_{gre}} (\lambda_{kret} - \lambda_{ge})^2 \right]^{\frac{1}{2}}, \tag{S7}$$

where M_{ge} is the number of barcode-replicate-time combinations at which λ_{kret} are estimated for background g in environment e. We estimate the fitness effects of mutations and their standard errors

$$\Delta\lambda_{mge} = \frac{1}{N_{mge}} \sum_{r,t} \sum_{k \in S_{mgr}} \Delta\lambda_{kret}, \tag{S8}$$

$$\sigma^{\Delta\lambda}_{mge} = \left[\frac{1}{M_{mge}(M_{mge}-1)} \sum_{r,t} \sum_{k \in S_{mgre}} (\Delta\lambda_{kret} - \Delta\lambda_{mge})^2 \right]^{\frac{1}{2}}, \tag{S9}$$

where M_{mge} is the number of barcode-replicate-time combinations at which $\Delta\lambda_{kret}$ are estimated for mutation m in background strain g and environment e.

The distributions of standard errors for background GRs and fitness effects are shown in Figure S2. The average standard error of the background GR is

$$\sigma_{\text{bg}} = 1.9 \times 10^{-3}\ \text{h}^{-1} \tag{S10}$$

and the average standard error of fitness effect is

$$\sigma_{\text{mut}} = 6.3 \times 10^{-3}\ \text{h}^{-1}. \tag{S11}$$

The comparison of these values shows that the noise in non-reference barcodes is typically more than 3-fold higher than noise in reference barcodes. Therefore, we note that, although equation (S9) ignores noise in reference barcodes, incorporating this noise would likely introduce only a small correction.

Detection of outlier barcodes. The goal of this procedure is to detect those barcodes whose frequencies either rise or fall unexpectedly quickly compared to the other barcodes tagging the same mutation in the same genetic background. To this end, we follow the method developed in Ref. (*3*), which takes as input the set of all barcodes k tagging a given mutation (in a given genetic background, biological replicate and environment), the corresponding cell counts N_{kt} at each time sampling t and the preliminary estimates $\widetilde{\Delta\lambda}_k$ of the fitness effects of all these barcoded lineages. Briefly, we calculate the "within-mutation" frequencies f_{kt} at time t as $f_{kt} = N_{kt}/\left(\sum_{k'} N_{k't}\right)$. All barcoded lineages tagging the same mutation should grow at the same rate. Therefore, we expect all frequencies f_{kt} to be constant over time, barring demographic and sampling noise. To determine which frequency trajectories are inconsistent

bioRxiv preprint doi: https://doi.org/10.1101/2023.11.18.567655; this version posted November 18, 2023. The copyright holder for this preprint (which was not certified by peer review) is the author/funder, who has granted bioRxiv a license to display the preprint in perpetuity. It is made available under aCC-BY 4.0 International license.

with this neutral expectation, we create a "within-mutation-neutral reference" (WMNR) set of barcodes whose preliminary fitness effect $\widetilde{\Delta\lambda}_k$ is within 0.01 of the median fitness effect of all these barcodes. Then, we fit two models to all frequency trajectories f_{kt} for those barcodes that are not in the WMNR set. In the neutral model, each query barcode's trajectory does not systematically change relative to the pooled WMNR trajectory. In the model with selection, query barcode's frequency can systematically increase or decrease. We find the log-likelihood of the observed barcode trajectory given each of the two models and calculate the likelihood ratio (LR) statistic. We conservatively exclude all barcodes with the LR statistic values greater than 40, corresponding to a P-value from a χ^2 distribution with 1 d.f. $< 10^{-9}$. This algorithm returns a "clean" subset of barcodes that tag a given mutation (in a given genetic background, environment and replicate).

Using this method, we exclude a total of 2.4% (16,799/683,754) of barcode-replicate combinations.

1.3.4 Calling beneficial and deleterious mutations

To call each mutation as either beneficial, deleterious or neutral in each genetic background and environment, we construct the 99% confidence interval using a normal distribution with the mean equal to $\Delta\lambda_{mge}$ (equation (S8)) and variance equal to the standard error of the mean $\sigma^{\Delta\lambda}_{mge}$ (equation (S9)). Mutations whose entire confidence interval is below zero are called deleterious and those whose entire confidence interval is above zero are called beneficial. All other mutations, i.e., those whose confidence interval spans zero, are called neutral. We identify a total of 7286 non-neutral mutation-genotype-environment combinations out of 18551 tested. If all mutations were truly neutral, we would expect to call 1% or ~ 185 of them non-neutral by chance, yielding the false discovery rate of $185/7286 = 2.5\%$.

1.3.5 Growth rates of background strains in YPD

Johnson et al (2019) estimated the mean, variance and skewness of tn-insertion DFEs in 163 yeast background strains g in rich YPD medium, as well as the fitness s_g of these strains relative to a common reference strain (*3*). They also separately measured exponential GR λ_g for a subset of their background strains. Using this subset of strains, we find a very good linear relationship between λ_g and s_g in YPD ($P = 9.632 \times 10^{-9}$, $R^2 = 0.97$),

$$\lambda_g = 0.9729\, s_g + 0.6732. \tag{S12}$$

We use equation (S12) to estimate the GR in YPD for all 163 strains.

1.3.6 Variation of growth rates and mutational effects across environments

To assess how the GR rank order of background strains varies across environments, we first find the median GR of all strains in each environment. We then call a strain as "above median" if its GR is above this median by at least one unit of its standard error σ^{λ}_{ge} (equation (S7)). Analogously, we call a strain as "below median" if its GR is below the median by at least σ^{λ}_{ge}. Any strain that is identified at least once as above median GR and at least once as below median GR was labelled as "Rank change" in Figure S6).

bioRxiv preprint doi: https://doi.org/10.1101/2023.11.18.567655; this version posted November 18, 2023. The copyright holder for this preprint (which was not certified by peer review) is the author/funder, who has granted bioRxiv a license to display the preprint in perpetuity. It is made available under aCC-BY 4.0 International license.

We assessed rank-order changes of mutations across environments within each strain using an analogous procedure (Figure S7).

1.3.7 Models of global epistasis

We fit equations (1) and (2) using `lm` function in R. To estimate the "pivot GR", we regress a_{me} against b_{me} (both estimated from the fit of (1)) with zero intercept and estimate pivot GR $\bar{\lambda}_e$ as the slope of this linear relationship.

Accounting for measurement errors in the calculation of global epistasis slopes. As pointed out by Berger and Postma (9), negative slopes in equation (1) (for any given mutation m and environment e) can arise spuriously due to fact that measurements errors in λ_{ge} and $\Delta\lambda_{mge}$ are correlated. Using the same approach as in Ref. (9), we compute the corrected correlation coefficient between $\Delta\lambda_{mge}$ and λ_{ge} across background genotypes for a fixed mutation m in a fixed environment e as

$$\rho'\left(\Delta\lambda_{mge}, \lambda_{ge}\right) = \frac{\mathrm{Cov}\left(\Delta\lambda_{mge}, \lambda_{ge}\right) + \sigma^2_{\mathrm{bg}}}{\sqrt{\left(\mathrm{Var}\left(\lambda_{ge}\right) - \sigma^2_{\mathrm{bg}}\right)\left(\mathrm{Var}\left(\Delta\lambda_{mge}\right) - \sigma^2_{\mathrm{bg}} - \sigma^2_{\mathrm{mut}}\right)}}. \tag{S13}$$

Here λ_{mge} and $\Delta\lambda_{mge}$ are given by equations (S6) and (S8), respectively; σ^2_{bg} and σ^2_{mut} are the measurement noise variance for GR of the background and mutant strains, respectively, which can be calculated from expressions (S10) and (S11), respectively; and

$$\begin{aligned}
\langle X \rangle &= \frac{1}{K}\sum_g X_g, \\
\mathrm{Var}\left(X\right) &= \frac{1}{K-1}\sum_g (X_g - \langle X \rangle)^2, \\
\mathrm{Cov}\left(X, Y\right) &= \frac{1}{K-1}\sum_g (X_g - \langle X \rangle)(Y_g - \langle Y \rangle).
\end{aligned}$$

are the estimates of the mean, variance and covariance, taken over all background genotypes g. We find that the uncorrected correlation coefficient

$$\rho\left(\Delta\lambda_{mge}, \lambda_{ge}\right) = \frac{\mathrm{Cov}\left(\Delta\lambda_{mge}, \lambda_{ge}\right)}{\sqrt{\mathrm{Var}\left(\lambda_{ge}\right)\,\mathrm{Var}\left(\Delta\lambda_{mge}\right)}}$$

deviates very little from the corrected correlation coefficient ρ' given by equation (S13) (see Figure S2D), indicating that the global epistasis trends we observe are not spurious.

Comparing distribution of slopes and intercepts across environments. We tested how the distributions of fitted slopes and intercepts vary across environments using three different pairwise tests (Figure S4).

1. A Kolmogorov–Smirnov test assess the overall differences between two distributions.

bioRxiv preprint doi: https://doi.org/10.1101/2023.11.18.567655; this version posted November 18, 2023. The copyright holder for this preprint (which was not certified by peer review) is the author/funder, who has granted bioRxiv a license to display the preprint in perpetuity. It is made available under aCC-BY 4.0 International license.

2. A paired t-test assess the differences between the means of the two distributions.

3. An F-test assess the difference between the variances of the two distributions.

All tests were performed using the stats package in R, and the raw P-values were adjusted using the Benjamini-Hochberg multiple testing correction. Adjusted P-values are reported in Figure S4A.

Comparing variable slopes and invariant slopes models for individual mutations. In addition to the full "variable slopes" model (equation (1)) in which a mutation can have different slopes in different environments, we also fit an "invariant slopes" model to our data in which every mutation has a single environment-invariant slope. We compared that variable and invariant slopes models using the likelihood ratio test and found that, for the majority of mutations, we could not reject the invariant slopes model in favor of the variable slopes model which has 5 more parameters (Figure S4C). We then calculated the adjusted R^2_{adj} for both the invariant and variable slopes models for each mutation using the R function `lm`,

$$R^2_{\text{adj}} = 1 - \left((1 - R^2) \frac{n-1}{n-k-1} \right),$$

where R^2 is the standard coefficient of determination, n is the number of observations and k is the number of predictors. In this case, n is the number of unique genotype-environment combinations in which the mutation is measured, and k is twice the number of environments in which the mutation is measured (variable slopes model) or the number of such environments plus one (invariant slopes model). This adjustment helps identify potential over-fitting by penalizing a high number of parameters relative to the number of observations.

Analysis of microscopic epistasis slopes. To determine whether global-epistasis slopes for a given mutation are statistically distinguishable across environments, we estimate these slopes as described above using the `lm` function in R. Along with the maximum likelihood estimates of the slopes, this function returns the standard errors of these estimates. Then, for each pairwise slope comparison, we calculate the difference between the slopes and estimate the associated error variance as the square root of the summed squared errors. Assuming that errors are normally distributed, we calculate the P-value for the observed error. We then apply the Benjamini-Hochberg multiple testing correction for all pairwise comparisons for a given mutation and obtain adjusted P-values. A pair of slopes is then called significantly different if the adjusted P-value is below 0.05.

1.3.8 Variance partitioning for the sign of mutations

Let Y_{mge} be the observed sign of mutation m in genetic background g in environment e, such that $Y_{mge} = \pm 1$. The total variance in the observed signs of mutations is

$$V^{\text{tot}} = \frac{1}{K-1} \sum_{m,g,e} \left(Y_{mge} - \overline{Y} \right)^2,$$

bioRxiv preprint doi: https://doi.org/10.1101/2023.11.18.567655; this version posted November 18, 2023. The copyright holder for this preprint (which was not certified by peer review) is the author/funder, who has granted bioRxiv a license to display the preprint in perpetuity. It is made available under aCC-BY 4.0 International license.

where

$$\overline{Y} = \frac{1}{K} \sum_{m,g,e} Y_{mge}$$

is the average sign of the mutational effect and $K = \sum_{m,g,e} 1$ is the total number of mutations measured across all genotypes and environments.

Let the true fitness effect of mutation m in genetic background g in environment e (without measurement noise) be

$$s_{mge} = G_{mge} + \xi_{mge},$$

where $G_{mge} = a_{me} + b_{me}\,\lambda_{ge}$ is the global epistasis term, λ_{ge} is the GR of background strain g in environment e, and ξ_{mge} is the idiosyncractic epistasis term (see equation (1)). Then, the probability that the observed sign of this mutation is positive is

$$p^{\mathrm{gl+id}}_{mge} = \Pr\left(Y_{mge} = 1\right) = \frac{1}{\sqrt{2\pi\sigma^2_{mge}}} \int_0^{\infty} \exp\left[-\frac{(x - s_{mge})^2}{2\sigma^2_{mge}}\right] dx, \qquad \text{(S14)}$$

where σ^2_{mge} is the variance of the measurements noise for mutation m in background g in environment e. The super-index $\mathrm{gl} + \mathrm{id}$ indicates that this probability takes into account both global and idiosyncratic epistasis. We estimate $p^{\mathrm{gl+id}}_{mge}$ using equation (S14) with s_{mge} and σ_{mge} given by equations (S8) and (S9). This allows us to calculate the expected sign of mutation m in background g in environment e,

$$\overline{Y}^{\mathrm{gl+id}}_{mge} = 2\,p^{\mathrm{gl+id}}_{mge} - 1$$

and estimate the variance in the mutational sign attributed solely to measurement noise,

$$V^{\mathrm{n}} = \frac{1}{K-1} \sum_{m,g,e} \left(Y_{mge} - \overline{Y}^{\mathrm{gl+id}}_{mge}\right)^2. \qquad \text{(S15)}$$

In a model without idiosyncratic epistasis, the probability p^{gl}_{mge} that the observed sign of the mutational effect is positive can be estimated using the same equation (S14), but with

$$\begin{aligned} s_{mge} &= G_{mge}, \\ \sigma^2_{mge} &= \frac{1}{M_{me}(M_{me}-1)} \sum_{r,t,g'} \sum_{k \in S_{mg're}} \left(\Delta\lambda_{kret} - G_{mg'e}\right)^2, \end{aligned}$$

where $\Delta\lambda_{kret}$ are estimated with equation (S2) and M_{me} is the number of barcode-genotype-replicate-time combinations at which $\Delta\lambda_{kret}$ are estimated for mutation m and environment e.

Thus, the variance in the mutational signs attributed to both idiosyncratic epistasis and measurement noise is

$$V^{\mathrm{id+n}} = \frac{1}{K-1} \sum_{m,g,e} \left(Y_{mge} - \overline{Y}^{\mathrm{gl}}_{mge}\right)^2,$$

where

$$\overline{Y}^{\mathrm{gl}}_{mge} = 2\,p^{\mathrm{gl}}_{mge} - 1$$

bioRxiv preprint doi: https://doi.org/10.1101/2023.11.18.567655; this version posted November 18, 2023. The copyright holder for this preprint (which was not certified by peer review) is the author/funder, who has granted bioRxiv a license to display the preprint in perpetuity. It is made available under aCC-BY 4.0 International license.

is the expected sign of mutation m in background g in environment e in the model without idiosyncratic epistasis. Thus, we can partition the total variance V^{t} into the global, idiosyncratic and noise components as follows. Variance V^{n} given by equation (S15) is attributed to noise, variance

$$V^{\mathrm{id}} = V^{\mathrm{id+n}} - V^{\mathrm{n}}$$

is attributed to idiosyncratic epistasis, and variance

$$V^{\mathrm{gl}} = V^{\mathrm{tot}} - V^{\mathrm{id+n}}$$

is attributed to global epistasis.

1.3.9 Empirical DFEs

For YPD, we use the estimates of DFE moments and their corresponding standard errors obtained in Ref. (3). In each other environment e and for each background strain g, we use the fitness effect estimates $\Delta\lambda_{mge}$ obtained from equation (S8) to estimate the mean $\langle\Delta\lambda\rangle_{ge}$, variance $\mathrm{Var}_{ge}\left[\Delta\lambda\right]$ and skewness $\mathrm{Skew}_{ge}\left[\Delta\lambda\right]$ of the empirical DFEs as

$$\begin{aligned}
\langle\Delta\lambda\rangle_{ge} &= \frac{1}{K_{ge}} \sum_m \Delta\lambda_{mge}, \\
\mathrm{Var}_{ge}\left[\Delta\lambda\right] &= \frac{1}{K_{ge}-1} \sum_m \left(\Delta\lambda_{mge} - \langle\Delta\lambda\rangle_{ge}\right)^2, \\
\mathrm{Skew}_{ge}\left[\Delta\lambda\right] &= \frac{\frac{1}{K_{ge}} \sum_m \left(\Delta\lambda_{mge} - \langle\Delta\lambda\rangle_{ge}\right)^3}{\left(\mathrm{Var}_{ge}\left[\Delta\lambda\right]\right)^{\frac{3}{2}}}.
\end{aligned}$$

Here, K_{ge} is the number of mutations whose effects were estimated in background strain g in environment e. To estimate the uncertainty in these estimates, we resampled 70 random mutations from each empirical DFE with replacement (bootstrapping). For each resampled mutation, we drew its fitness effect from a normal distribution with mean $\Delta\lambda_{mge}$ (given by equation S8) and standard deviation $\sigma^{\Delta\lambda}_{mge}$ (given by equation S9). We performed 300 iterations of this procedure.

Sensitivity of DFE moment estimates to missing measurements. In pooled cultures, slow growing mutants may go extinct during the competition assay, which could prevent us from estimating their effects and lead to biases in our estimates of the DFE moments. In particular, highly deleterious mutations missing from the data could leads us to overestimate the DFE mean, underestimate the DFE variance and overestimate DFE skewness. Furthermore, we expect that these biases would be stronger in slower growing background strains, which could produce spurious declines in DFE mean and skewness with the background-strain GR.

As described above, we sought to mitigate this potential issue experimentally by competing our mutants in groups with similar GRs (see Section 1.2.3). However, these spurious effects may still be present in our data. To investigate how severe these effects might be, we plotted the number of mutations for which we have reliable fitness-effect estimates against the respective

bioRxiv preprint doi: https://doi.org/10.1101/2023.11.18.567655; this version posted November 18, 2023. The copyright holder for this preprint (which was not certified by peer review) is the author/funder, who has granted bioRxiv a license to display the preprint in perpetuity. It is made available under aCC-BY 4.0 International license.

background strain GR. We found that the number of mutations per strain varies little with the background-strain GR in 30°C environments (Figure S10B), but it does vary in the expected direction in 37°C environments. However, since this analysis does reveal the fitness effects of missing mutations, this observation alone does not imply that our estimates of DFE moments are biased for slow-growing strains in 37°C environments. Thus, we carried out two additional analyses to further probe probe the extent of these potential biases.

First, we eliminated all strains from our analysis for which we measured less than 60 mutations (bottom 24%) and replotted DFE moments for this reduced number of strains (Figure S10A) and found that all the trends found in the full data set remain in this reduced data set (compare Figure S10C and 4). Second, in each environment, we identified the set of 40 mutations all of which were measured in the maximum number of strains in that environment, which ranged from 16 to 40 strains, depending on environment. We then restricted our analysis of DFE moments to these strains and mutations, thereby creating a reduced data set without any missing measurements. We found that the DFE moments recapitulate the trends reported for the full data set (compare Figures S10D and 4). Based on these analyses, we conclude that the trends in the DFE moments that we report are not spurious results of missing measurements.

Variation of the DFEs with adjusted GR. We carried a series of pairwise DFE comparisons across strains and/or environments. To this end, we created matched pairs of background strains using three methods:

1. *Adjusted GR matching.* We matched each background strain g_1 in environment e_1 with another background strain $g_2 \neq g_1$ in a different environment $e_2 \neq e_1$, such that the adjusted GR $\lambda^*_{g_2 e_2}$ of the latter strain was most similar to the former strain among all other strain-environment combinations.

2. *Raw GR matching.* We carried out the same procedure as above except we matched raw GRs.

3. *Strain matching.* We matched each background strain g_1 in environment e_1 with itself in another environment e_2 such that $\lambda_{g_1 e_2}$ was the closes to $\lambda_{g_1 e_1}$.

Then, we compared the DFEs of the two matched strain-environment combinations using four metrics of similarity shown in Figure S9.

1.3.10 QTL analysis

With only 42 background strains, we have little power to identify QTLs *de novo*. Instead, we assess whether certain loci identified in previous studies (*2*, *3*) help explain some of the idiosyncratic epistasis. Specifically, four loci Chr XIV-376315 (KRE33), ChrXII-646707, ChrXIV-470303, and ChrXV-154799 were identified as having significant explanatory power on the fitness effect of multiple mutations by both Jerison et al (2017) (Ref. (*2*)) and Johnson et al (2019) (Ref. (*3*), and we tested whether these candidate loci also have significant explanatory power in our data. We used ANOVA to determine whether the addition of these four loci to the generalized global epistasis model (equation (2)) significantly reduced the proportion of unexplained variance for

bioRxiv preprint doi: https://doi.org/10.1101/2023.11.18.567655; this version posted November 18, 2023. The copyright holder for this preprint (which was not certified by peer review) is the author/funder, who has granted bioRxiv a license to display the preprint in perpetuity. It is made available under aCC-BY 4.0 International license.

each mutation. If the contribution of a locus was significant at P-value 0.05, then we also calculated the fraction of variance explained by this locus above and beyond the generalized global epistasis model.

Out of a total of 89 mutations in which our models explained some variance in their fitness effects, we found two mutations (in genes UBP3 (ID 95) and STH1 (ID 41)) where 38% and 21% of overall variance was jointly explained by the four candidate QTLs, corresponding to 59% and 27% of the explained variance in each mutation, respectively. In one additional mutation, nearby HO (ID 78), these candidate loci together explain 8% of the overall variance in its effect, but this comprises 100% of the explained variance. For the remaining 97% (86/89) mutations, the four candidate loci together explain less than 14% of overall variance, with a median of only 3% (interquartile interval [1.8%, 5.7%]), corresponding to a median of 6.8% of the total explained variance within each mutation (interquartile interval [3.9%, 15.6%], Supplementary Table S4-Tab 10).

1.4 Theoretical calculation of DFE moments

Here we derive the moments of the distribution of fitness effects (DFE) of mutations from the generalized global epistasis equation (equation (2) in the main text). Since we consider the environment fixed, we drop the subindex e. To simplify notations, we will denote the adjusted GR of the background strain g by $F_g \equiv \lambda_g^*$ and we denote the fitness effect of a mutation in this background by $s_g \equiv \Delta\lambda_g$. To derive DFE moments, we assume that the effects of mutations s are drawn from a continuous distribution defined by equation

$$s_g = b\, F_g + \eta + \xi_g, \tag{S16}$$

which is the continuous analog of equation (2). Here b and η are the slope and the y-intercept of the focal mutation, and ξ_g is the idiosyncratic epistasis of this mutation in the background strain g. We assume that b and η are independent, and that b has probability density $p_{\rm sl}$, η is normally distributed with zero mean and variance $\sigma^2_{\rm pivot}$. We also assume that ξ_g is normally distributed with zero mean and variance $\sigma^2_{\rm id}$ (which can in principle depend on b, see Ref. (10)). Thus, conditional on b and η, the fitness effects s_g of the mutation in the background g is a normal random variable with distribution with mean $\langle s|b,\eta\rangle = b\, F_g + \eta$ and variance $\sigma^2_{\rm id}$. Then, the DFE $p_g(s)$ in the background g with adjusted GR F_g is given by

$$p_g(s) = \int_{-\infty}^{\infty} db\, p_{\rm sl}(b) \int_{-\infty}^{\infty} d\eta\, N\left(\eta; 0, \sigma^2_{\rm pivot}\right)\, N\left(s; b\, F_g + \eta, \sigma^2_{\rm id}\right), \tag{S17}$$

where $N(x;\mu,\sigma^2)$ is the normal probability density with mean m and variance σ^2. Since

$$N\left(s; b\, F_g + \eta, \sigma^2_{\rm id}\right) = N\left(\eta; s - b\, F_g, \sigma^2_{\rm id}\right)$$

and since

$$N\left(x;\mu_1,\sigma_1^2\right)\, N\left(x;\mu_2,\sigma_2^2\right) = \frac{1}{\sqrt{2\pi\left(\sigma_1^2+\sigma_2^2\right)}} \exp\left(-\frac{(\mu_1-\mu_2)^2}{2\left(\sigma_1^2+\sigma_2^2\right)}\right) N(x,\mu_3,\sigma_3^2),$$

bioRxiv preprint doi: https://doi.org/10.1101/2023.11.18.567655; this version posted November 18, 2023. The copyright holder for this preprint (which was not certified by peer review) is the author/funder, who has granted bioRxiv a license to display the preprint in perpetuity. It is made available under aCC-BY 4.0 International license.

where $\mu_3 = \frac{\sigma_1^{-2}}{\sigma_1^{-2}+\sigma_2^{-2}}\,\mu_1 + \frac{\sigma_2^{-2}}{\sigma_1^{-2}+\sigma_2^{-2}}\,\mu_2$ and $\sigma_3^2 = \frac{1}{\sigma_1^{-2}+\sigma_2^{-2}}$ for any μ_1, μ_2, σ_1^2 and σ_2^2, the integral with respect to η can be taken, such that the expression (S17) simplifies to

$$p_g(s) = \int_{-\infty}^{\infty} db\, p_{\rm sl}(b)\, N\left(s; b\, F_g, \tilde{\sigma}^2\right), \tag{S18}$$

where we denoted $\tilde{\sigma}^2 = \sigma^2_{\rm pivot} + \sigma^2_{\rm id}$.

Equation (S18) allows us to compute the DFE mean as

$$\langle s \rangle = \int_{-\infty}^{\infty} db\, p_{\rm sl}(b) \int_{-\infty}^{\infty} ds\, s\, N\left(s; b\, F_g, \tilde{\sigma}^2\right) = \langle b \rangle\, F_g \tag{S19}$$

and higher central moments of the DFE as

$$M^{(n)}\left[s\right] = \int_{-\infty}^{\infty} db\, p_{\rm sl}(b) \int_{-\infty}^{\infty} ds\, \left(s - \langle b \rangle\, F_g\right)^n\, N\left(s; b\, F_g, \tilde{\sigma}^2\right) \tag{S20}$$

Using expression (S20) and the fact that

$$s - \langle b \rangle\, F_g = \left(s - bF_g\right) + \left(b - \langle b \rangle\right) F_g$$

we obtain the following explicit expressions for the DFE variance $\mathrm{Var}\left[s\right]$ and its third central moment $M^{(3)}\left[s\right]$,

$$\begin{aligned}
\mathrm{Var}\left[s\right] &\equiv M^{(2)}\left[s\right] = \int_{-\infty}^{\infty} db\, p_{\rm sl}(b) \left[\tilde{\sigma}^2 + \left(b - \langle b \rangle\right)^2 F_g^2\right] \\
&= \mathrm{Var}\left[b\right]\, F_g^2 + \sigma^2_{\rm pivot} + \int_{-\infty}^{\infty} p_{\rm sl}(b)\, \sigma^2_{\rm id}\, db, \qquad \text{(S21)}\\
M^{(3)}\left[s\right] &= \int_{-\infty}^{\infty} db\, p_{\rm sl}(b) \left[3\tilde{\sigma}^2 \left(b - \langle b \rangle\right) F_g + \left(b - \langle b \rangle\right)^3 F_g^3\right] \\
&= M^{(3)}\left[b\right]\, F_g^3 + 3F_g \int_{-\infty}^{\infty} p_{\rm sl}(b)\, \sigma^2_{\rm id}\, \left(b - \langle b \rangle\right)\, db. \qquad \text{(S22)}
\end{aligned}$$

Here $\mathrm{Var}\left[b\right]$ and $M^{(3)}\left[b\right]$ are the variance and the third central moment of the distribution $p_{\rm sl}(b)$ of global epistasis slopes, respectively.

We find that the variance of residuals and slopes are correlated (Figure S4C). Thus, we set $\sigma^2_{\rm id} = -\alpha\, b$, and the expressions (S21) and (S22) become

$$\mathrm{Var}\left[s\right] = -\alpha\, \langle b \rangle + \sigma^2_{\rm pivot} + \mathrm{Var}\left[b\right]\, F_g^2, \tag{S23}$$

$$M_g^{(3)}\left[s\right] = -3\alpha\, \mathrm{Var}\left[b\right]\, F_g + M^{(3)}\left[b\right]\, F_g^3. \tag{S24}$$

and the skewness of the DFE is given by

$$\mathrm{Skew}_g\left[s\right] = \frac{M_g^{(3)}\left[s\right]}{\left(\mathrm{Var}_g\left[s\right]\right)^{3/2}} = \frac{-3\alpha\, F_g/\sqrt{\mathrm{Var}\left[b\right]} + \mathrm{Skew}\left[b\right]\, F_g^3}{\left(\frac{-\alpha\, \langle b \rangle + \sigma^2_{\rm pivot}}{\mathrm{Var}[b]} + F_g^2\right)^{3/2}}. \tag{S25}$$

bioRxiv preprint doi: https://doi.org/10.1101/2023.11.18.567655; this version posted November 18, 2023. The copyright holder for this preprint (which was not certified by peer review) is the author/funder, who has granted bioRxiv a license to display the preprint in perpetuity. It is made available under aCC-BY 4.0 International license.

Equations (S19), (S23) and (S25) show that when $F_g = 0$, DFE mean and skewness are zero and the DFE variance achieves its minimal value σ^2_{id}. Furthermore, since $\text{Skew}\,[b] < 0$, DFE skewness monotonically declines from $-\text{Skew}\,[b] > 0$ to $\text{Skew}\,[b] < 0$.

To show that all odd moments of the DFE vanish when $F_g = 0$, we notice that, when $F_g = 0$, equation (S18) simplifies to

$$p_g(s) = N\left(s; 0, \tilde{\sigma}^2\right), \tag{S26}$$

i.e., the DFE is a normal distribution, which implies that all its odd central moments vanish. To show that all even moments of the DFE reach their minimum at $F_g = 0$, we differentiate expression (S20) with respect to F_g at $F_g = 0$ and obtain

$$\left.\frac{dM^{(n)}[s]}{dF}\right|_{F_g=0} = \int_{-\infty}^{\infty} db\, p_{\text{sl}}(b)\left[-n\langle b\rangle M^{(n-1)}[s|b] + \frac{b}{\tilde{\sigma}^2} M^{(n+1)}[s|b]\right].$$

where $M^{(n)}[s|b] = \int_{-\infty}^{\infty} s^n N\left(s; 0, \tilde{\sigma}^2\right) ds$ is the nth central moment of a normal distribution with mean zero and variance $\tilde{\sigma}^2$. Therefore, when n is even, $\left.\frac{dM^{(n)}[s]}{dF}\right|_{F_g=0}$ vanishes because all odd central moments of a normal distribution are zero. To see that $M^{(n)}[s]$ achieve their minimum at $F_g = 0$ for any even n, we find that the second derivative of $M^{(n)}[s]$ at $F_g = 0$ is given by

$$\left.\frac{d^2M^{(2k)}[s]}{dF^2}\right|_{F_g=0}$$
$$= \int_{-\infty}^{\infty} db\, p_{\text{sl}}(b)\left[n(n-1)\langle b\rangle^2 M^{(n-2)}[s|b] - \frac{b}{\tilde{\sigma}^2}\left(b + 2n\langle b\rangle\right) M^{(n)}[s|b] + \frac{b^2}{\tilde{\sigma}^4} M^{(n+2)}[s|b]\right].$$

The even moments $M^{(n)}[s|b]$ of the normal distribution $N\left(s; 0, \tilde{\sigma}^2\right)$ can be expressed as $\tilde{\sigma}^n(n-1)!!$ where $(n-1)!! = (n-1)(n-3)\cdots 3\cdot 1$ is the double factorial. Therefore, we have

$$\left.\frac{d^2M^{(2k)}[s]}{dF^2}\right|_{F_g=0} = (n-1)!!\, n \int_{-\infty}^{\infty} p_{\text{sl}}(b)\, \tilde{\sigma}^{n-2}\left(b - \langle b\rangle\right)^2 db > 0$$

for any even n, which implies that $M^{(2k)}[s]$ indeed achieves its minimum at $F_g = 0$.

bioRxiv preprint doi: https://doi.org/10.1101/2023.11.18.567655; this version posted November 18, 2023. The copyright holder for this preprint (which was not certified by peer review) is the author/funder, who has granted bioRxiv a license to display the preprint in perpetuity. It is made available under aCC-BY 4.0 International license.

References

1. Bloom JS, Ehrenreich IM, Loo WT, Lite TLV, Kruglyak L. Finding the sources of missing heritability in a yeast cross. Nature. 2013;494(7436):234–237.

2. Jerison ER, Kryazhimskiy S, Mitchell JK, Bloom JS, Kruglyak L, Desai MM. Genetic variation in adaptability and pleiotropy in budding yeast. Elife. 2017;6:e27167.

3. Johnson MS, Martsul A, Kryazhimskiy S, Desai MM. Higher-fitness yeast genotypes are less robust to deleterious mutations. Science. 2019;366(6464):490–493.

4. Johnson MS, Desai MM. Mutational robustness changes during long-term adaptation in laboratory budding yeast populations. Elife. 2022;11:e76491.

5. Wrolstad R, Acerr T, Decker E, Penner M, Reid D, Schwartz S, et al. Common buffers and stock solutions. Current Protocols in Food Analytical Chemistry, John Wiley & Sons, Inc. 2001;.

6. Gomori G. Preparation of buffers for use in enzyme studies. Biochemistry and Molecular Biology. 2010; p. 719.

7. Levy SF, Blundell JR, Venkataram S, Petrov DA, Fisher DS, Sherlock G. Quantitative evolutionary dynamics using high-resolution lineage tracking. Nature. 2015;519(7542):181–186.

8. Venkataram S, Kinsler G. BarcodeCounter2; 2021. https://github.com/sandeepvenkataram/BarcodeCounter2.

9. Berger D, Postma E. Biased estimates of diminishing-returns epistasis? Empirical evidence revisited. Genetics. 2014;198(4):1417–1420.

10. Reddy G, Desai MM. Global epistasis emerges from a generic model of a complex trait. Elife. 2021;10:e64740.

bioRxiv preprint doi: https://doi.org/10.1101/2023.11.18.567655; this version posted November 18, 2023. The copyright holder for this preprint (which was not certified by peer review) is the author/funder, who has granted bioRxiv a license to display the preprint in perpetuity. It is made available under aCC-BY 4.0 International license.

2 Supplementary tables

**Environment**		**Slopes**			**Intercepts**		
Temp, °C	pH	Mean	Stdev	Skew	Mean	Stdev	Skew
30	3.2	−0.109	0.127	−1.492	0.023	0.029	0.724
30	5.0	−0.202	0.185	−1.154	0.059	0.054	0.442
30	7.0	−0.142	0.164	−0.867	0.026	0.035	0.325
37	3.2	−0.133	0.151	−1.015	0.03	0.039	0.45
37	5.0	−0.112	0.162	−0.872	0.025	0.05	0.46
37	7.0	−0.135	0.172	−0.755	0.016	0.036	−0.816

Table S1. Statistics of slope and intercept distributions.

bioRxiv preprint doi: https://doi.org/10.1101/2023.11.18.567655; this version posted November 18, 2023. The copyright holder for this preprint (which was not certified by peer review) is the author/funder, who has granted bioRxiv a license to display the preprint in perpetuity. It is made available under aCC-BY 4.0 International license.

**Antibiotic**	***E. coli***	***S. cerevisiae***
Kanamycin (Kan)	40	N/A
Ampicilin (Amp)	100	100
Nourseothricin (Nat)	20	20
Hygromycin (Hyg)	200	300

Table S2. Antibiotic concentrations used in this study, in µg/ml.

bioRxiv preprint doi: https://doi.org/10.1101/2023.11.18.567655; this version posted November 18, 2023. The copyright holder for this preprint (which was not certified by peer review) is the author/funder, who has granted bioRxiv a license to display the preprint in perpetuity. It is made available under aCC-BY 4.0 International license.

3 Supplementary figures

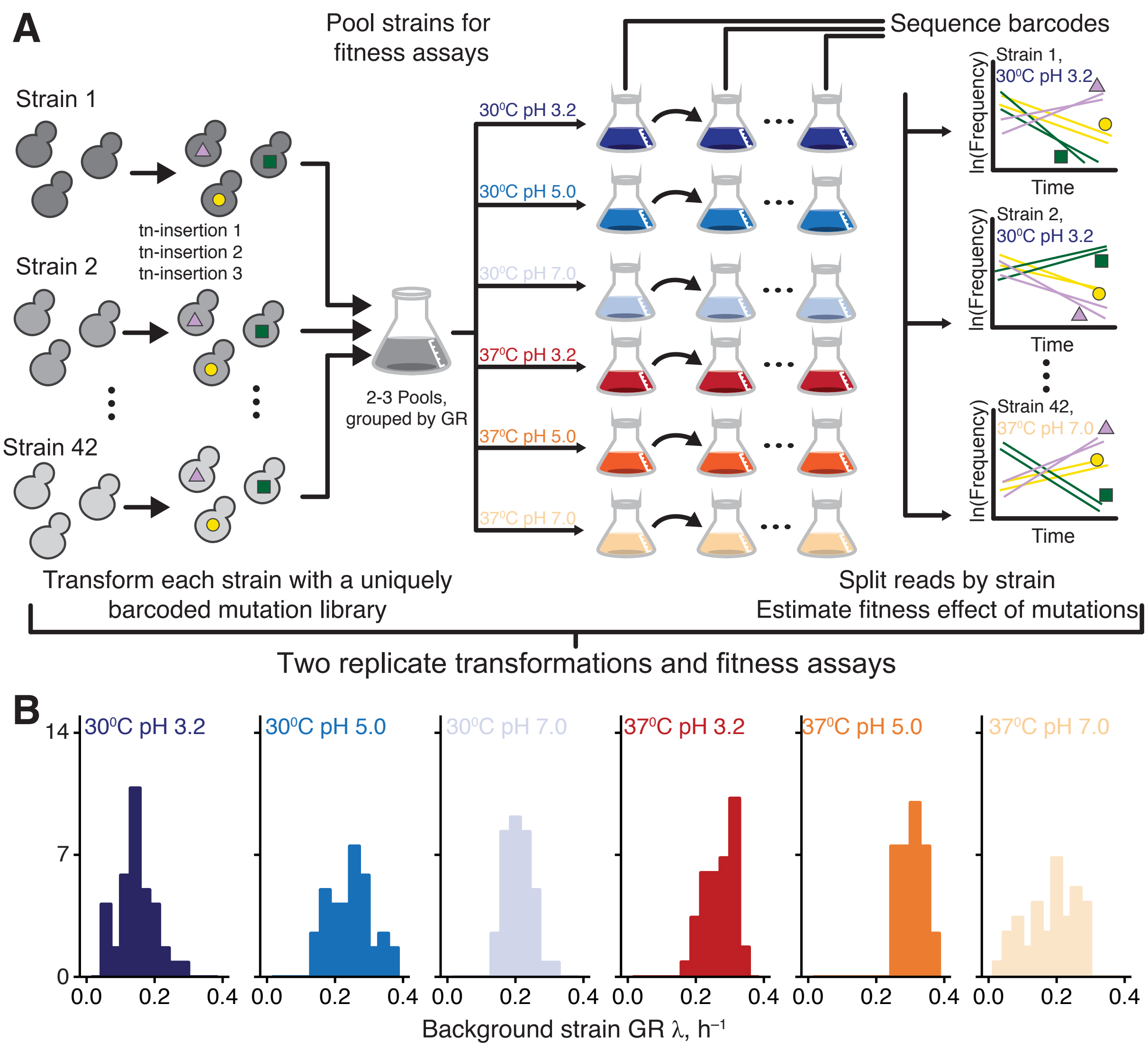

Figure S1. Experimental setup. A. Schematic of the experiment. **B.** Distribution of GRs of background strains in all environments.

bioRxiv preprint doi: https://doi.org/10.1101/2023.11.18.567655; this version posted November 18, 2023. The copyright holder for this preprint (which was not certified by peer review) is the author/funder, who has granted bioRxiv a license to display the preprint in perpetuity. It is made available under aCC-BY 4.0 International license.

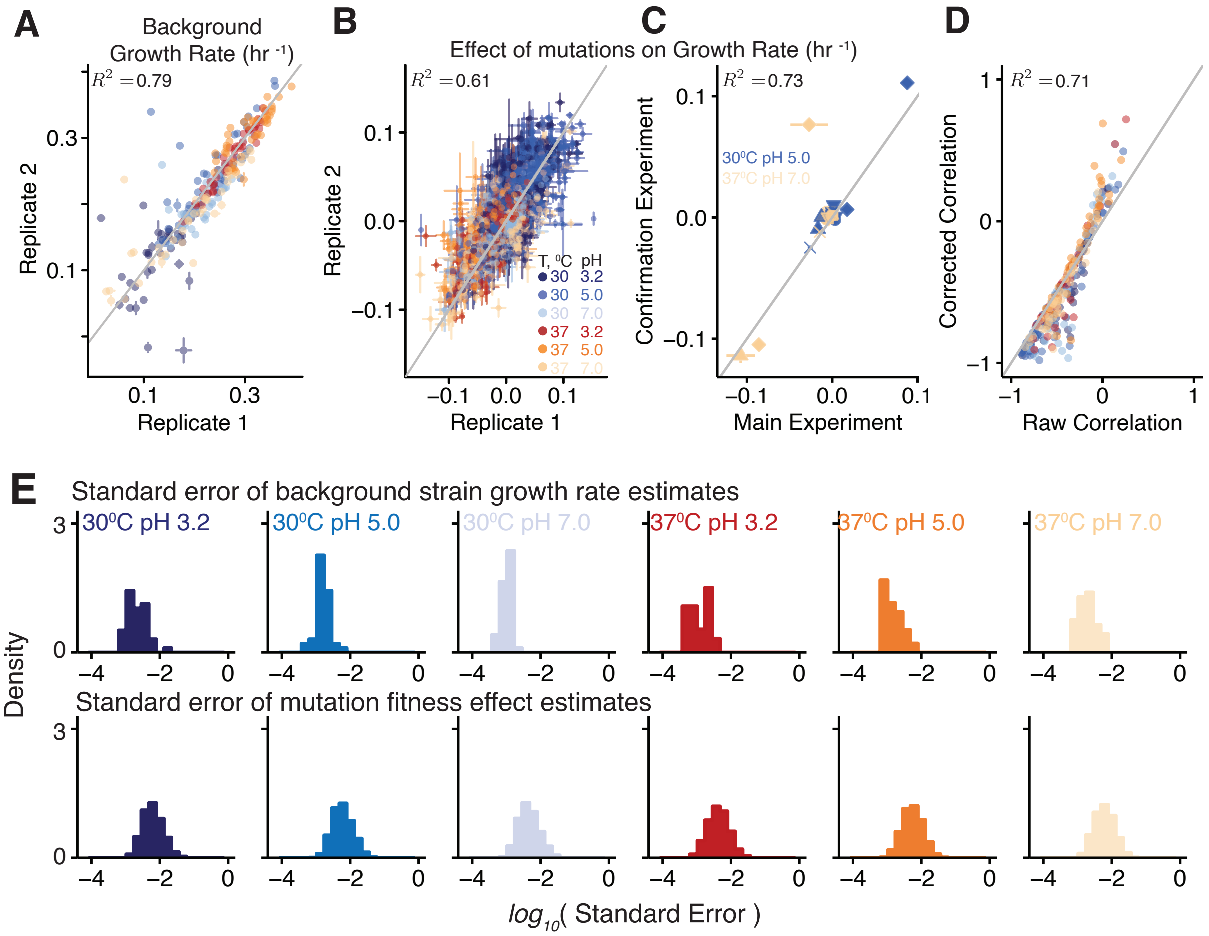

Figure S2. Data quality checks. A. Correlation between GRs of background strains estimated in two biological replicates. **B.** As in panel A, but for the fitness effects of mutations. In both panels, error bars represent ±1 standard error. **C.** Correlation between fitness effect estimates in the high-throughput RB-TnSeq experiment and the validation experiment (see Section 1.2.3). **D.** Raw and corrected estimates of the correlation coefficient between background GR and fitness effect for each mutation in each environment. In all panels, grey line is the diagonal, R^2 is reported for linear regression ($P < 0.01$ for all regressions). **E.** Distribution of the standard error of background strain GRs (top) and mutation effects (bottom) in all environments.

bioRxiv preprint doi: https://doi.org/10.1101/2023.11.18.567655; this version posted November 18, 2023. The copyright holder for this preprint (which was not certified by peer review) is the author/funder, who has granted bioRxiv a license to display the preprint in perpetuity. It is made available under aCC-BY 4.0 International license.

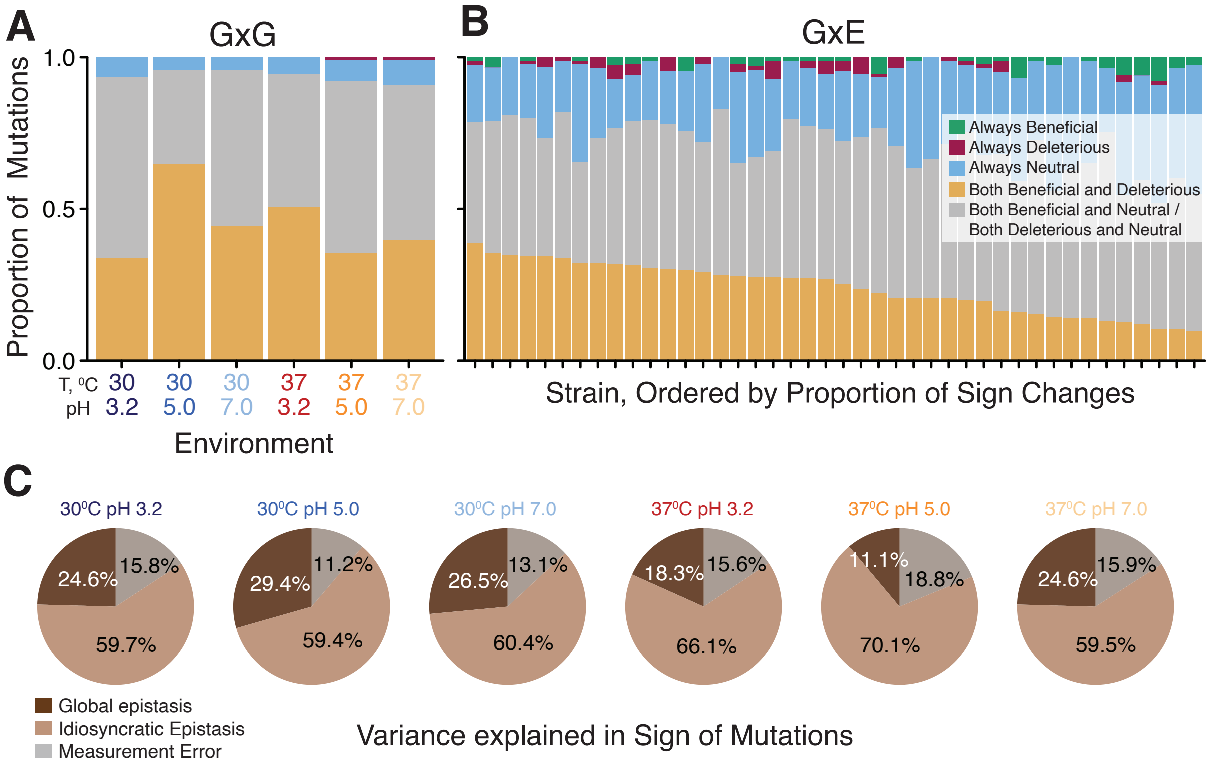

Figure S3. Variation in the sign of mutational effects across genetic backgrounds (G×G interactions) and across environments (G×E interactions). A. Proportions of mutations that do and do not change sign across background strains in each environment. **B.** Proportions of mutations that do and do not change sign across environments in each background strain. **C.** The proportion of variance in the observed sign of mutations in each environment explained by measurement noise, global and idiosyncratic epistasis (see Section 1.3.8 for details).

bioRxiv preprint doi: https://doi.org/10.1101/2023.11.18.567655; this version posted November 18, 2023. The copyright holder for this preprint (which was not certified by peer review) is the author/funder, who has granted bioRxiv a license to display the preprint in perpetuity. It is made available under aCC-BY 4.0 International license.

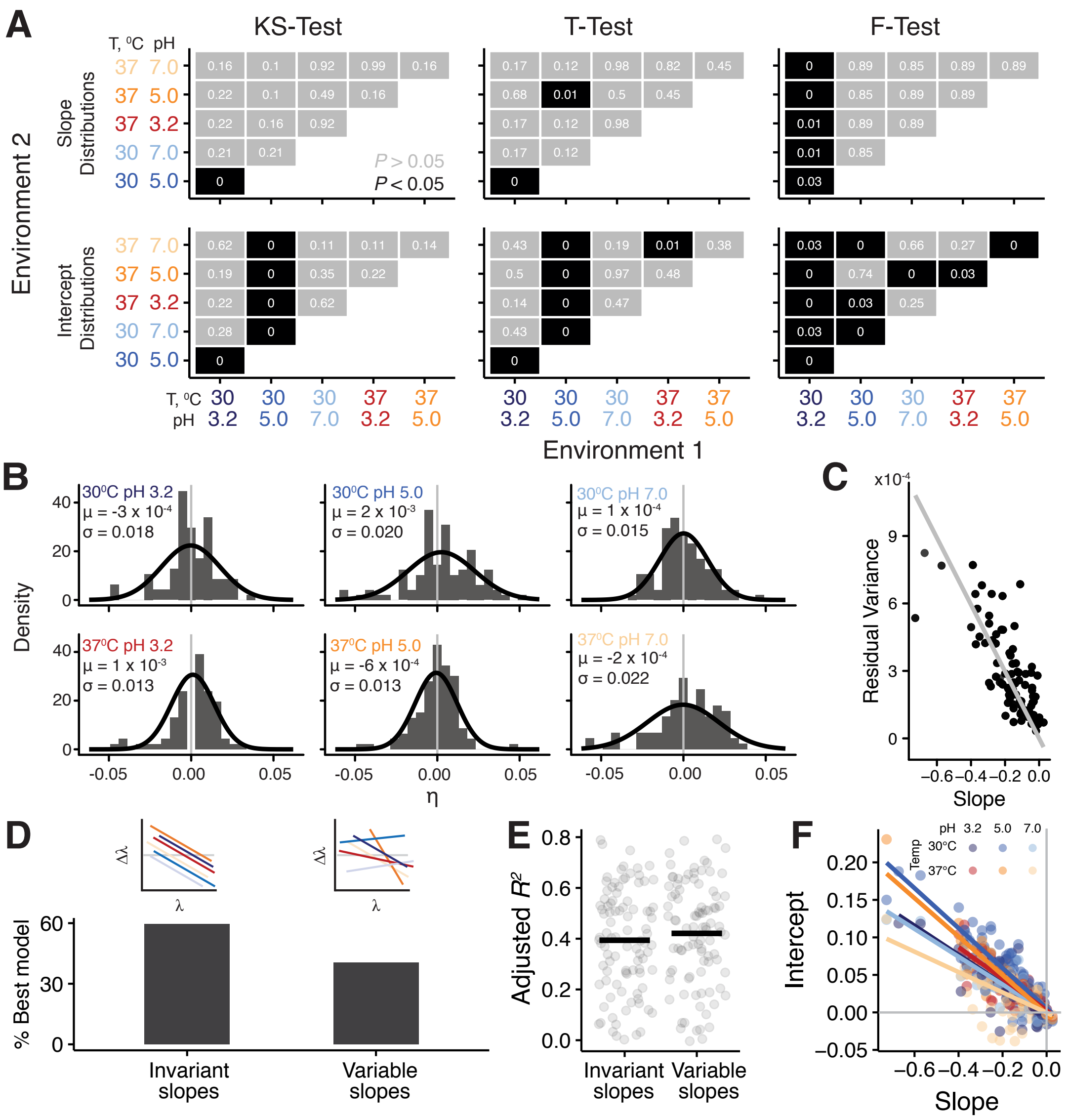

Figure S4. Properties of global epistasis models A. Comparison of distributions of slopes (top) and intercepts (bottom) across environments using three metrics (see Section 1.3.7. The number in each tile is the P-value (after Benjamini-Hochberg correction) of the comparison, and tiles with $P < 0.05$ are colored black. **B.** Distribution of the pivot noise term η in each environment. Mean and variance of the distribution are labelled in each panel, and the best fit normal distribution is overlayed. **C.** Relationship between global epistasis slope and the variance of residuals from the fit of equation (2). Grey line is best fit linear regression through the origin. **D.** Bar graph showing the percent of mutations best fit by the invariant slopes and variable slopes models. Illustrative example of each model is shown on top. **E.** The adjusted R^2 for the fits of both models for all mutations. **F.** Slope intercept correlation for a fit of the invariant slopes model, lines are best fit linear regression through the origin.

bioRxiv preprint doi: https://doi.org/10.1101/2023.11.18.567655; this version posted November 18, 2023. The copyright holder for this preprint (which was not certified by peer review) is the author/funder, who has granted bioRxiv a license to display the preprint in perpetuity. It is made available under aCC-BY 4.0 International license.

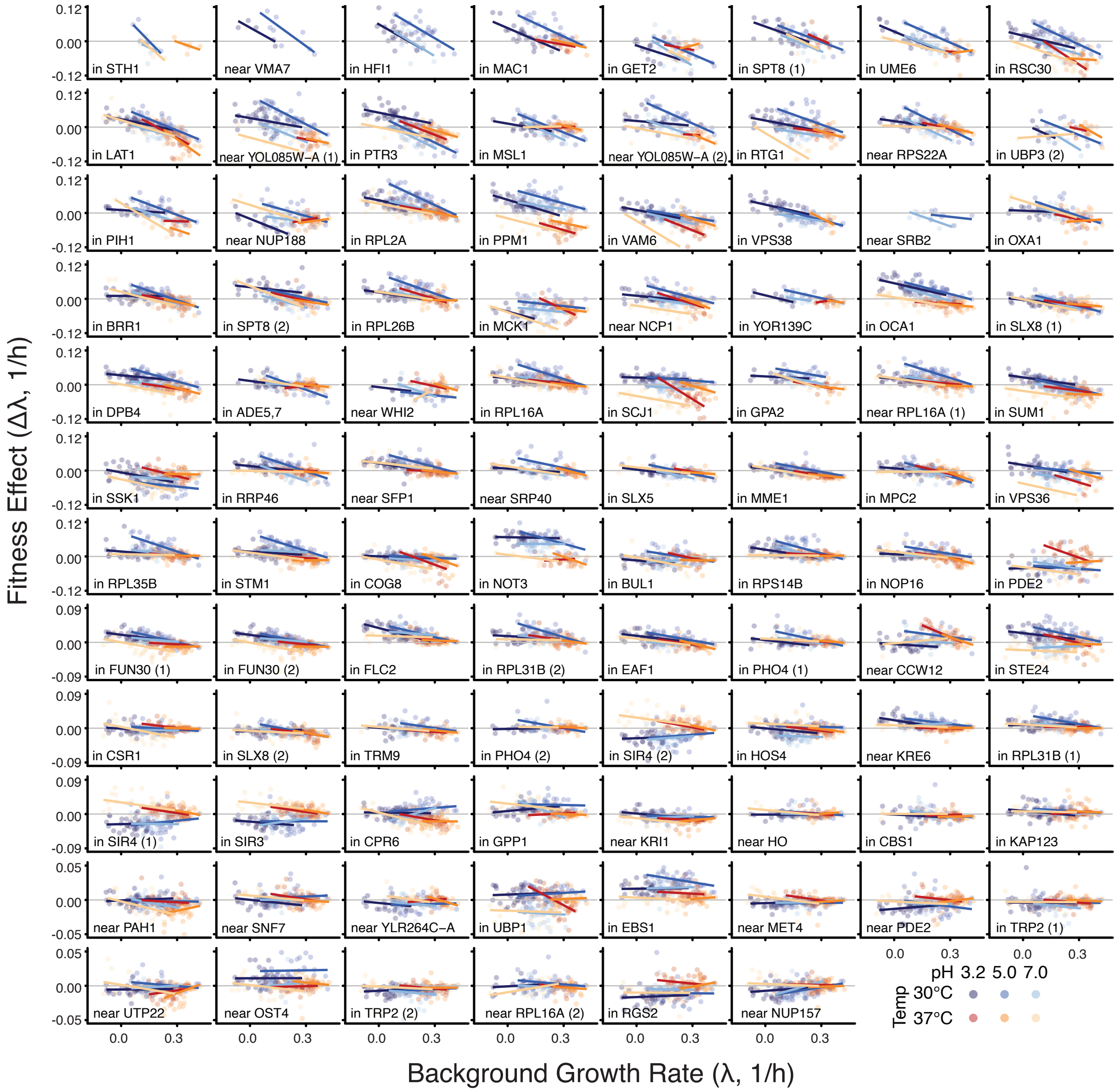

Figure S5. Global epistasis model for individual mutations. Same as Figure 3, but with data points. Mutations are ordered by slope, as in Figure 3. y-axis varies across rows.

bioRxiv preprint doi: https://doi.org/10.1101/2023.11.18.567655; this version posted November 18, 2023. The copyright holder for this preprint (which was not certified by peer review) is the author/funder, who has granted bioRxiv a license to display the preprint in perpetuity. It is made available under aCC-BY 4.0 International license.

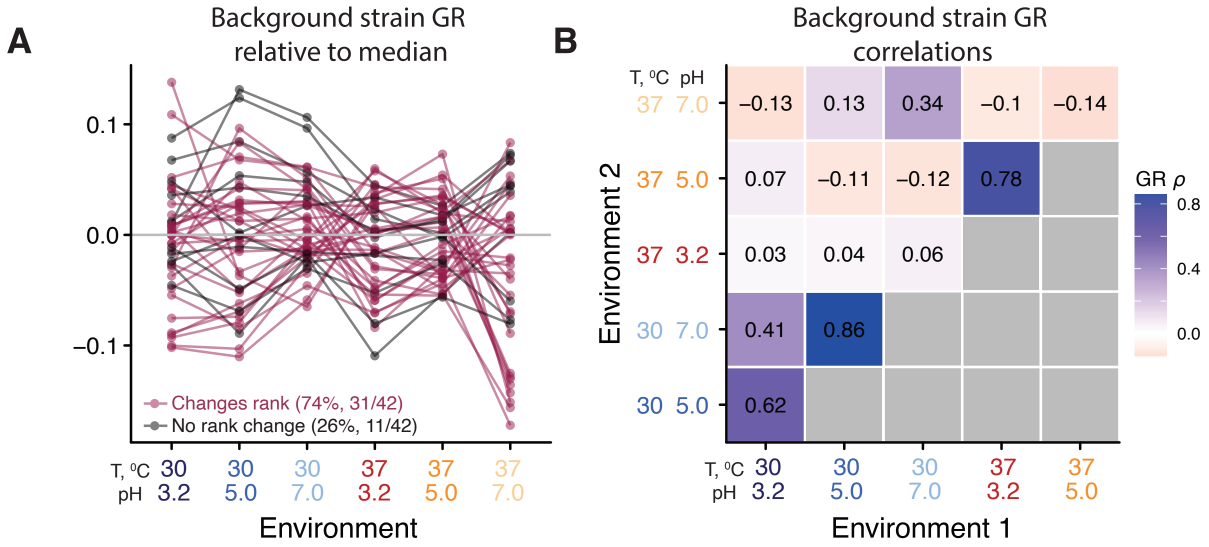

Figure S6. Reshuffling of background strain GRs across environments. A. Strain GR relative to the median GR in each environment. Lines connect the same strain across environments and are colored maroon if the strain is on different sides of the median in different environments. **B.** The correlation of background strain GR across all pairs of environments.

bioRxiv preprint doi: https://doi.org/10.1101/2023.11.18.567655; this version posted November 18, 2023. The copyright holder for this preprint (which was not certified by peer review) is the author/funder, who has granted bioRxiv a license to display the preprint in perpetuity. It is made available under aCC-BY 4.0 International license.

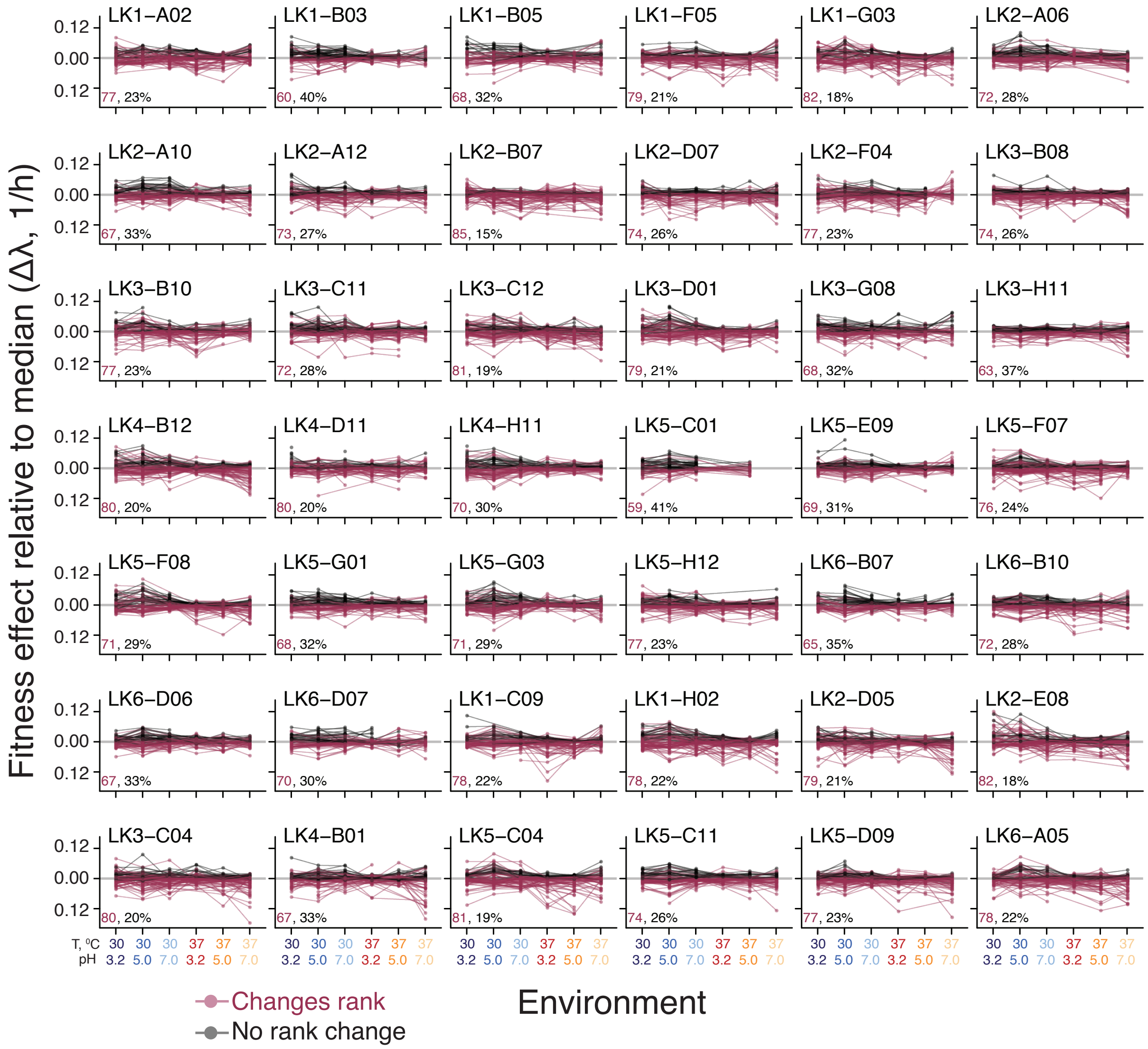

Figure S7. Reshuffling of the effects of mutations across environments. Each panel corresponds to a background strain and shows the effect of all mutations relative to the median in each environment. Lines connect the same mutation across environments and are colored maroon if the mutation is on different sides of the median in different environments.

bioRxiv preprint doi: https://doi.org/10.1101/2023.11.18.567655; this version posted November 18, 2023. The copyright holder for this preprint (which was not certified by peer review) is the author/funder, who has granted bioRxiv a license to display the preprint in perpetuity. It is made available under aCC-BY 4.0 International license.

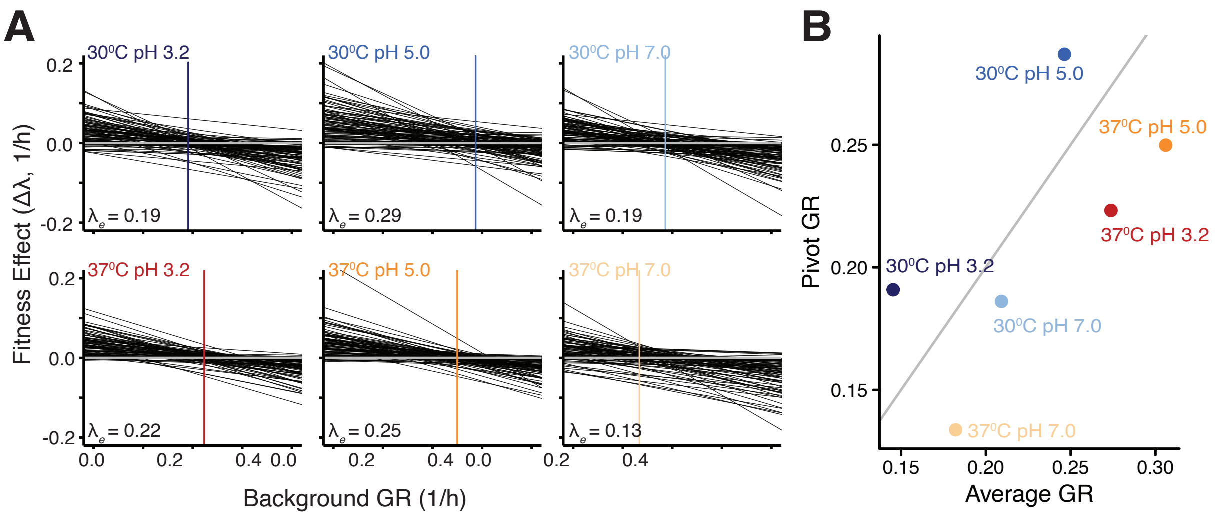

Figure S8. Pivot GRs across environments. A. Best-fit regression lines of the generalized global epistasis equation (2)for all mutations in each environment. Vertical line represents the pivot GR λ_e. **B.** Relationship between the mean background strain GR in each environment and the pivot GR.

bioRxiv preprint doi: https://doi.org/10.1101/2023.11.18.567655; this version posted November 18, 2023. The copyright holder for this preprint (which was not certified by peer review) is the author/funder, who has granted bioRxiv a license to display the preprint in perpetuity. It is made available under aCC-BY 4.0 International license.

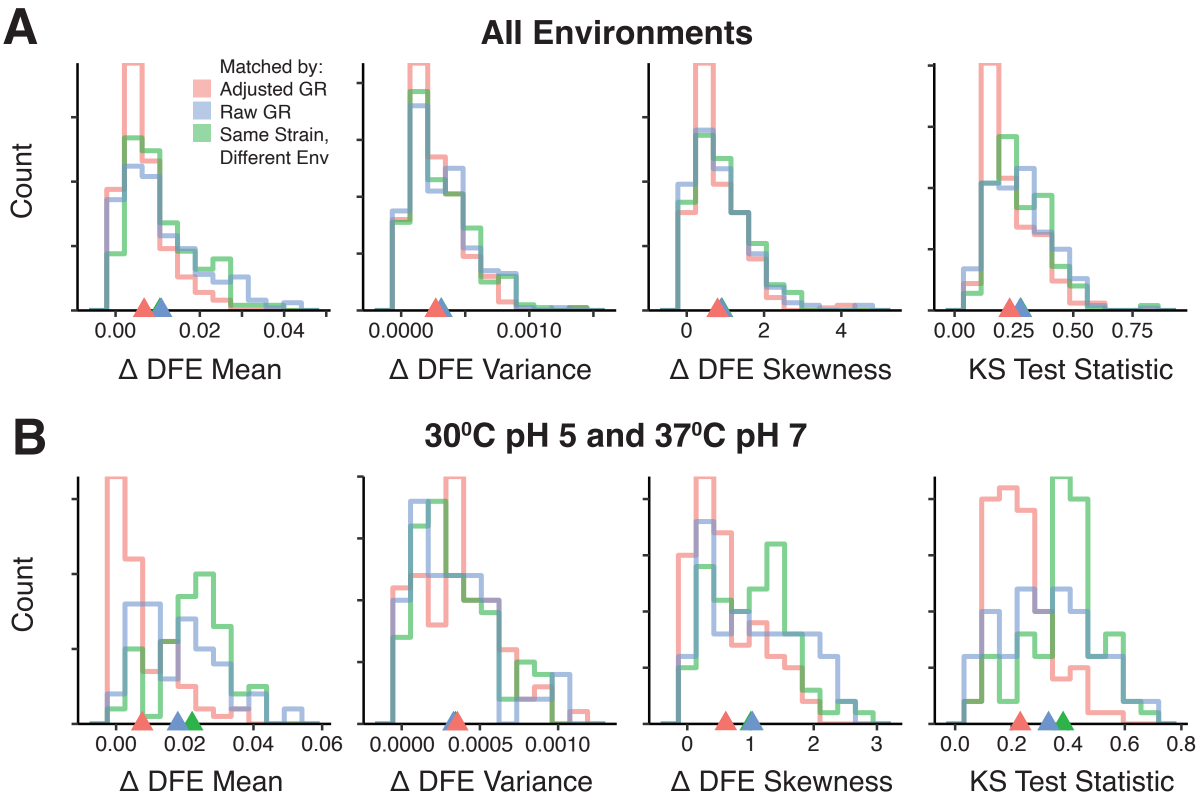

Figure S9. Distributions of DFEs similarity statistics. A. Distribution of four metrics of DFE similarity for all pairs of strains from any two different environments and matched either by their adjusted GR (red), raw GR (blue), or strain identity (green). See Section 1.3.9 for details. For all metrics, lower values mean more similar DFEs. Triangle shows the mean of the corresponding colored distribution. **B.** Same as A but with the strains sampled from the two most dissimilar environments, 30°C pH 5 and 37°C pH 7.

bioRxiv preprint doi: https://doi.org/10.1101/2023.11.18.567655; this version posted November 18, 2023. The copyright holder for this preprint (which was not certified by peer review) is the author/funder, who has granted bioRxiv a license to display the preprint in perpetuity. It is made available under aCC-BY 4.0 International license.

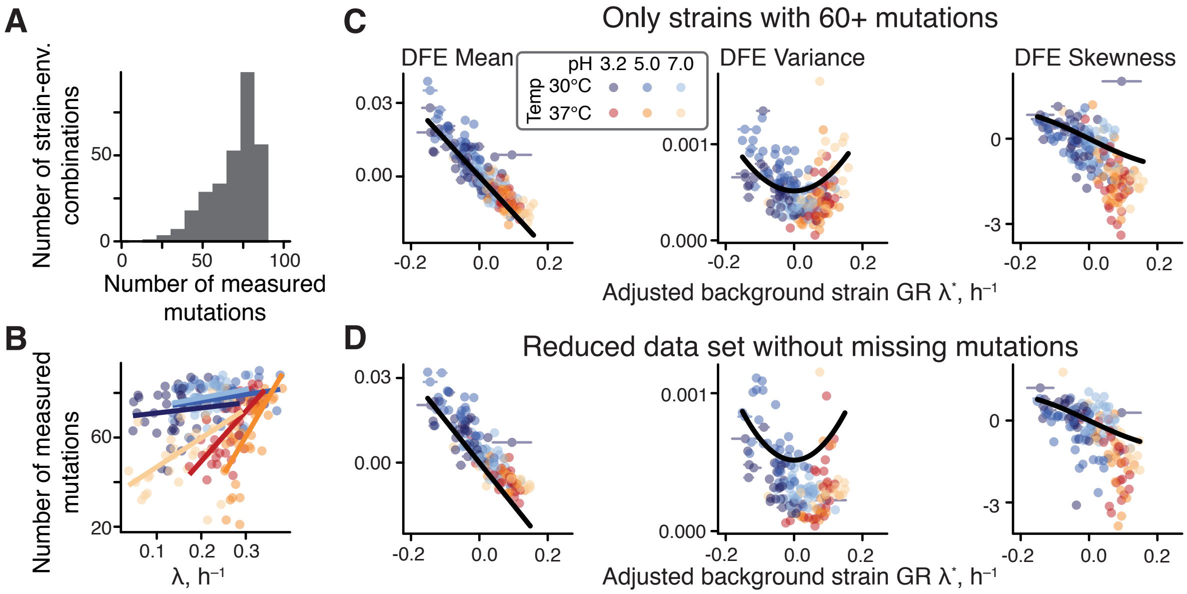

Figure S10. Robustness of the observed DFE variation with respect to missing measurements. A. Distribution of the number of mutations measured per DFE. **B.** Relationship between the number of mutations in each DFE and the background GR. Lines represent the best fit linear regression. **C.** Same as Figure 4 but excluding all strains whose DFE contains less than 60 mutations. **D.** Same as Figure 4 but based on a reduced data set without missing measurements (see Section 1.3.9).

bioRxiv preprint doi: https://doi.org/10.1101/2023.11.18.567655; this version posted November 18, 2023. The copyright holder for this preprint (which was not certified by peer review) is the author/funder, who has granted bioRxiv a license to display the preprint in perpetuity. It is made available under aCC-BY 4.0 International license.

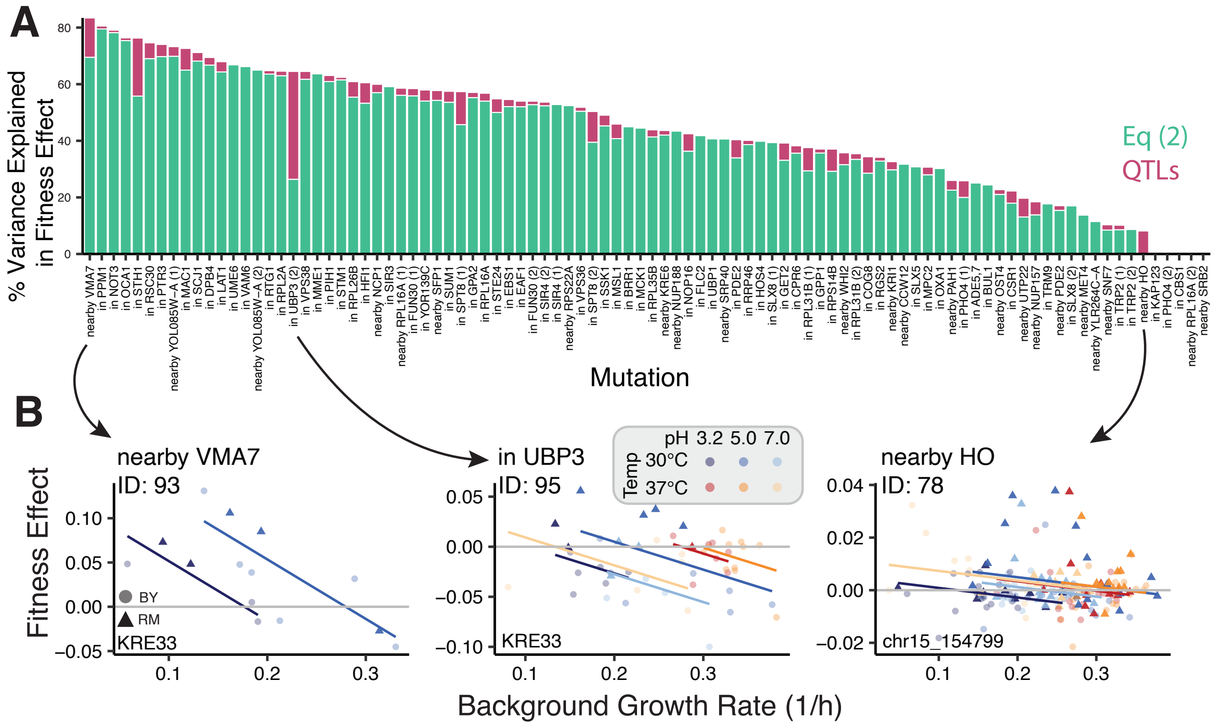

Figure S11. QTL analysis. A. The percent variance in fitness effect of each mutation explained by four candidate loci combined (pink) above and beyond the variance explained by the generalized global epistasis equation (2)(teal). Mutations are ordered by the total explained variance. **B.** Three example mutations, with lines representing the best fit generalized global epistasis model, colored by environment. Point shape represents the allele, either BY (circles) or RM (triangles), at the locus explaining most variation for that mutation (locus indicated in the bottom left of each panel).
